# Phylogenetic Analysis of the Genes in D-Ala-D-Lactate Synthesizing Glycopeptide Resistance Operons: The Different Origins of Functional and Regulatory Genes

**DOI:** 10.3390/antibiotics13070573

**Published:** 2024-06-21

**Authors:** Gábor Kardos, Levente Laczkó, Eszter Kaszab, Bálint Timmer, Krisztina Szarka, Eszter Prépost, Krisztián Bányai

**Affiliations:** 1 Institute of Metagenomics, University of Debrecen, H-4032 Debrecen, Hungary; timmer.balint@pte.hu (B.T.); szkrisz@med.unideb.hu (K.S.); 2 One Health Institute, Faculty of Health Sciences, University of Debrecen, H-4032 Debrecen, Hungary; laczko.levente@med.unideb.hu (L.L.); kaszab.eszter@univet.hu (E.K.); 3 HUN-REN-UD Conservation Biology Research Group, H-4032 Debrecen, Hungary; 4 Department of Microbiology and Infectious Diseases, University of Veterinary Medicine, H-1078 Budapest, Hungary; 5 Department of Medical Microbiology and Immunology, University of Pécs, H-7624 Pécs, Hungary; 6 Department of Health Industry, University of Debrecen, H-4032 Debrecen, Hungary; prepost.eszter@unideb.hu; 7 Pathogen Discovery Group, HUN-REN Veterinary Medical Research Institute, H-1143 Budapest, Hungary; 8 National Laboratory for Infectious Animal Diseases, Antimicrobial Resistance, Veterinary Public Health and Food Chain Safety, H-1143 Budapest, Hungary; 9 Department of Pharmacology and Toxicology, University of Veterinary Medicine, H-1078 Budapest, Hungary

**Keywords:** phylogeny, antimicrobial resistance, *Streptomyces*, *Paenibacillus*

## Abstract

The phylogenetic relationships of glycopeptide resistance proteins were investigated. The amino acid sequences of vanA, vanB, vanR and vanS were used as queries to search against bacterial genomes in the NCBI RefSeq database. Hits with >60% amino acid identity and >90% query coverage were aligned, and phylogenetic trees were reconstructed. The ligase gene phylogenies were highly similar for both queries, revealing two major clusters. One contained [[vanA:vanM][vanB:vanD]vanF] and related proteins, with proteins from different Bacillaceae, mostly from *Paenibacillus* spp., in basal positions to all, except vanB. Ligases from streptomycetes formed the other cluster. The relative positions of vanH and vanX differed from those of the associated ligases, but the basal position of the *Paenibacillus* spp. and the separation of proteins of *Streptomyces* origin were similar. The accessory genes vanW, vanY and vanZ were associated with vanB, vanA/vanM and vanA, respectively; the basal branches were always proteins from different Bacillaceae but never from streptomycetes. Multiple homologs of the regulatory genes vanR and vanS were found in the genomes; those associated with the different ligases were unique to the ligases. Similarly to the accessory genes, vanRS from Bacillales and Clostridia, but never from streptomycetes, was found in the basal positions. In conclusion, the core genes vanA/B/D/F/M, vanH and vanX originate most probably from glycopeptide-producing streptomycetes, with *Paenibacillus* spp. (or other Bacillaceae) mediating the transfer, while the accessory genes and the regulatory apparatus probably originate from these Bacillaceae.

## 1. Introduction

Vancomycin is a glycopeptide antibiotic produced by *Amycolatopsis orientalis* (formerly *Streptomyces orientalis*) discovered in 1953 in a soil sample from Borneo [1]. Later, several natural compounds with a similar structure were found, such as avoparcin and teicoplanin, which were introduced into veterinary and human use, respectively [1]. These drugs gained importance in human therapy when extensive resistance to beta-lactam drugs appeared in Gram-positive bacteria and serve as salvage drugs against methicillin-resistant *Staphylococcus aureus* and against enterococci resistant to beta-lactams, especially against *Enterococcus faecium* [2,3,4,5]. In parallel, glycopeptides, primarily vancomycin, became a frequent component of empirical therapeutic regimens for serious infections. For this reason, resistance to glycopeptides is of great concern, and vancomycin-resistant enterococci are considered one of the main public health threats in the ESKAPE group.

Glycopeptides bind to the D-Ala-D-Ala end of the pentapeptide involved in transpeptidation and are steric inhibitors of the process [1,2,3]. This binding is mediated by several hydrogen bonds between the alanines and glycopeptides, which are crucial to antibacterial action. Resistance is mediated mainly by alteration of the pentapeptide target, disrupting the hydrogen bonds involved in drug binding [3]. Two such altered pentapeptide types were found ending with either D-Ala-D-Ser, conferring a moderate level of vancomycin resistance, or with D-Ala-D-lactate, which usually results in a high level of resistance [3].

The D-Ala-D-Lac operons are typically composed of ’core’ genes, which are essential to vancomycin resistance, and a set of ‘accessory’ genes, which appear to have a non-essential role. The core gene cassette is divided into a set of regulatory genes and a set of genes responsible for the synthesis of D-Ala-D-Lac. For example, in the vanA operon, vanR and vanS constitute the regulatory gene cassette, whereas the vanHAX cassette consists of a dehydrogenase (vanH), a ligase (vanA) and a D,D-dipeptidase (vanX), while the components of the accessory gene cassette are a D,D-carboxypeptidase (vanY) and a gene whose product is of an unknown function (vanZ). Other D-Ala-D-Lac operons show different degrees of structural variation.

In fact, the resistance mechanisms are mediated by several vancomycin resistance operons, which have been named after the ligases responsible for the formation of the dipeptide/depsipeptide end of the pentapeptide but usually also contain enzymes responsible for eliminating the D-Ala-D-Ala dipeptide associated with glycopeptide susceptibility and for providing the (amino) acid incorporated into the novel dipeptide/depsipeptide. The operons vanA and vanB are the most important clinically, both producing pentapeptides with a D-lactate terminus and thus conferring high-level resistance [2,3,4,5]. Both occur commonly in *E. faecium* and *E. faecalis* and less frequently in other *Enterococcus* species, termed vancomycin-resistant enterococci (VRE), which cause significant clinical concern. Rarely, they have been found in methicillin-resistant *Staphylococcus aureus* as well.

Despite their public health importance, there are gaps in the knowledge of the phylogeny and origin of these genes/operons. The aim of this study was to analyze their occurrence and to elucidate the phylogenetic relationships among the operons responsible for glycopeptide resistance.

## 2. Results

### 2.1. Resistance Ligases (vanA, vanB, vanD, vanF, vanM)

**Figure 1:**
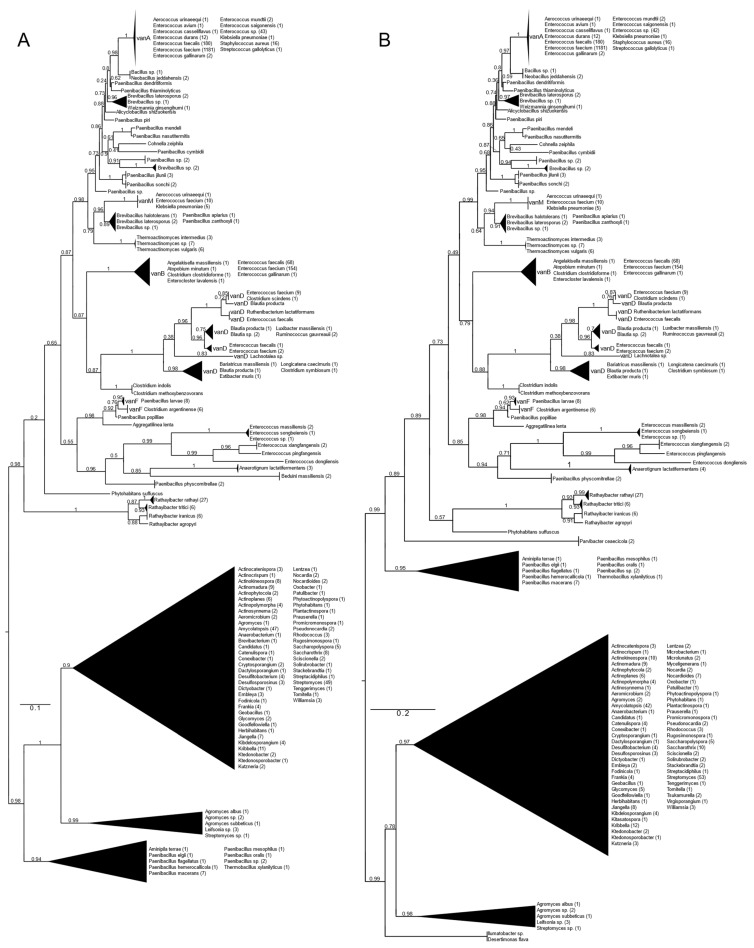
Phylogenetic tree of D-Ala-D-lactate ligases as reconstructed based on vanA (GenBank Accession number: KR047792; panel (**A**) and vanB (GenBank Accession number: KC489787; panel (**B**) queries after filtering BLAST hits for 60% aa identity and ≥90% coverage. Clades were collapsed to reduce redundancy in the tree. Roots are placed based on the result of MAD 2.2. Support values refer to the SH-like local support calculated by FastTree. For the full phylogenetic trees and MAD root placement probabilities, see Supplementary Materials (Figure S1).

All types of D-alanyl-D-lactate ligases (vanA, vanB, vanD, vanF, vanM) were found with vanA as well as with vanB as a query (Figure 1 and Figure S1). With vanA and vanB as the queries, phylogenetic trees with practically the same topology of the major branches were obtained. They differed in the relative position of the *Paenibacillus* clade (*P. macerans*, *P. elgii*, *P. hemerocallicola*), which formed a sister group to all the BLAST hits of the glycopeptide producers with vanA as the query, while with vanB, the *Paenibacillus* clade was closer to the crown group of the vancomycin resistance ligases (see below). 

Rooting with minimum ancestor deviation identified two well-supported major clusters: one contained the vanA, vanB, vanM, vanD and vanF proteins and proteins related to these, while the other contained the D-ala-D-lactate ligases from *Streptomyces* and relatives (Figure 1 and Figure S1).

When using vanA as a query, within the stem group of the large vanA-vanM-vanB-vanD-vanF clade, multiple proteins could be identified from different *Rathayibacter* spp., as well as *Phytohabitans suffuscus*. Using a vanB query, several *Paenibacillus* sequences closely related to *Aminipila terrae* and *Thermobacillus xylanilyticus* were the most basal group of this clade, followed by the diversification of *Parvibacter caecicola*.

The first branching separated vanF and its related proteins. The clade of *Enterococcus massiliensis*, *E. songbeiensis*, *E. xiangfangensis*, *E. pingfangensis*, *E. dongliensis*, *Anaerotignum lactatifermentans* and *Paenibacillus physcomitrellae* appeared to be a sister to vanF found in *Aggregatilinea lenta*, *Paenibacillus popilliae*, *Clostridium argentinense* and *Paenibacillus larvae*. However, the basal position of this clade to all the vanA, vanM, vanB and vanD proteins was not unequivocal. The next branch grouped vanB and vanD as sister clades. The clade vanB contained proteins from *E. faecium*, *E. faecalis*, *E. gallinarum*, *Atopobium minutum*, *Enterocloster lavalensis*, *Clostridium clostridioforme* and *Angelakisella massiliensis*, forming three distinguishable subclusters. Curiously, there were no other taxa closely related to the vanB clade besides the cluster containing vanD, which appeared to be a sister to vanB. Proteins from *Clostridium symbiosum*, *C. indolis* and *C. methoxybenzovorans*, rather than *Lachnotalea*, *Bariatricus*, *Blautia*, *Extibacter* and *Longicatena*, could be identified as the stem of the vanD clade. The crown group of vanD contained proteins from *E. faecium*, *E. faecalis*, *Clostridium scindens*, *Blautia* spp., *Ruthenibacterium*, *Ruminococcus* and *Luxibacter*.

The vanM clade (*E. faecium*, *Aerococcus urinaeequi* and *K. pneumoniae*) was a sister to a group consisting of *Brevibacillus laterosporus*, *Paenibacillus apiarus*, *P. halotolerans* and *P. zanthoxyli*. Curiously, one of the sequences from *E. faecium* (accession no. GCF_009734005) corresponded to the genome harboring vanP. *Thermoactinomycetes* spp. could be identified as the stem of this clade, although with a lower support value.

A grade consisting of *Paenibacillus*, *Brevibacillus*, *Alicyclobacillus* and *Cohnella* could be identified toward the crown clade of vanA. Within this grade, *Paenibacillus* diverged the earliest. As sisters to *Neobacillus* and *Bacillus*, the vanA proteins formed a monophyletic cluster and were highly homogeneous among enterococci and were present in *E. faecium*, *E. faecalis*, *E. mundtii*, *E. avium*, *E. gallinarum*, *E. saigonensis*, *E. casseliflavus*, *Aerococcus urinaeequi*, *Streptococcus gallolyticus*, *Staphylococcus aureus* and *Klebsiella pneumoniae*.

The other major clade contained proteins from various streptomycetes, including the glycopeptide producers (*Actinoplanes teichomyceticus*, *Amycolatopsis orientalis*, *Streptomyces tohoensis*), as well as others from various related species.

Analysis of the split vanA and vanB proteins showed that all fragments were closest to the *Enterococcus* genus.

### 2.2. vanH

When vanH was used as the query, two main sister clusters were delineated (Figure S2). One consisted of poorly resolved clusters of proteins from streptomycetes, including various glycopeptide producers, and proteins from various soil-associated Firmicutes and Actinobacteria. The earliest branch contained proteins from various *Paenibacillus* spp. (e.g., *P. elgii*: vanRSWHAX, absent in other *P. elgii* genomes), while another early diverged branch included L-lactate-dehydrogenases from *Lentzea* and *Dactylosporangium*.

The stem of the other sister group was a protein from *Phytohabitans suffuscus*; then, it was split into two subclusters. The first contained two main groups, one of proteins from thermoactinomycetes, sister to a group of proteins from *Clostridium argentinense*, *Paenibacillus popilliae*, *P. physcomitrellae* and *Aggregatilinea lenta*. The other group within this clade included sequences from *Enterococcus faecium* with vanM (vanRSYHM on a plasmid), *Aerococcus urinaeequi* and *Klebsiella* spp., with proteins from *Brevibacillus* spp., *Paenibacillus apiarius* and *P. zanthoxyli* as the most closely related sequences.

Within the first branch of the other subcluster, proteins from *Lachnotalea*, *Lacrimispora* and *Clostridium methoxybenzovorans* were found in the basal position, and the remaining BLAST hits identified highly similar proteins from *E. faecium* and *E. faecalis* (vanB-type), *E. gallinarum* (carrying vanA and vanB), *Angelakisella massiliensis* (vanRSYHBX on the chromosome), *Enterocloster clostridioforme* (vanRSYWHBX), *E. lavalensis* (vanRSYWHBX) and *Atopobium minutum* (vanRSYHBX on the chromosome in a genetic environment very similar to that found in *Angelakisella*), as well as the vanD-associated vanH proteins in a separated clade with a well-resolved structure (((*Ruminococcus*, *Blautia*, *E. faecium*, *E. faecalis*, *Ruthenibacterium*) *Luxibacter*); (*E. faecium*, *Clostridium scindens*)); ((*Longicatena*, *C. symbiosum*) *Extibacter muris*)). The stem taxa in the other main branch were various *Paenibacillus* spp., *Neobacillus*, *Brevibacillus*, *Alicyclobacillus*, *Cohnella zeiphila* and *Weizmannia ginsengihumi*, of which the most basal were various *Paenibacillus* species. The sister group of these contained the vanA-associated vanH from *E. faecalis*, *E. faecium*, *E. saigonensis*, *S. aureus*, *Aerococcus urinaaeequi*, *E. casseliflavus*, *E. avium*, *E. gallinarum*, *E. durans*, *E. mundtii* and *K. pneumoniae*.

### 2.3. vanX

Two major clades were distinguished: one contained vanX from *Streptomyces* and relatives, including the glycopeptide producers, while the other contained all the vanX proteins from organisms with the D-Ala-D-lactate ligases involved in acquired glycopeptide resistance (Figure S3). Within the latter clade, *Phytohabitans suffuscus* could be separated first, similarly to with vanH, followed by the separation of *Paenibacillus* spp. and *Thermobacillus xylanilyticus*. The next branch contained the vanX-D group (species as for the crown of vanD), together with a possible but poorly supported sister group of proteins from *Aminipila terrae*, *Longicataena caecimuris*, *Adlercreutzia mucosicola*, *Enterorhabdus caecimuris*, *Parvibacter caecicola* and *Enterocloster clostridioformis*.

A clade sister to this group containing vanX-D proteins contained vanX proteins associated with other ligases (vanA, vanB, vanM and vanF); the basal branches separated with low support values included vanX from *Beduini massiliensis*, *Anaerotignum lactatifermentans* and *Paenibacillus physcomitrellae*. In the next well-supported branch were proteins from *Thermoactinomyces* spp., *Aggregatilinea lenta*, *Paenibacillus popilliae* (vanRSYZHFX) and *Clostridium argentinense*, as well as all the vanX-A, vanX-B, vanX-M and vanX-F proteins. However, the structure within was poorly resolved, showing uncertainty in the relatedness of the vanX proteins associated with different ligases. 

Within this group, the most basal in the vanX-B group was a single *Paenibacillus apiarius* sequence, followed by the separation of a group of proteins from *Paenibacillus* spp. and *Cohnella zeiphila*. The next branch immediately basal to the vanX-Bs consisted of vanX proteins from *C. indolis* and *C. methoxybenzovorans*. The vanX-B protein sequences were divided into three groups ((proteins from *E. faecium* and *E. faecalis*, *Angelakisella massiliensis*, *Enterocloster clostridioforme*, *Atopobium minutum*); (*E. faecium* and *E. faecalis*, *E. gallinarum*); (*E. faecium* and *E. faecalis*, *Enterocloster clostridioforme*)). 

The position of the vanX-M proteins was uncertain, while proteins from *Weizmannia ginsengihumi*, *Brevibacillus* spp., *Paenibacillus zanthoxyli* and *P. thiaminolyticus* were basal to the vanX-A proteins.

### 2.4. vanW

The protein vanW was found to be linked to the vanB operon only; the stem group is a well-structured group of ((*Paenibacillus mendelii*, *P. nasutitermis*); (*Brevibacillus* spp., *P. piri*) (*Clostridium indolis*, *C. methoxybenzovorans*, *Paenibacillus* spp.) (Figure S4). The next branch comprised two related but well-separable groups: (*E. faecalis*, *E. faecium*) and (*E. faecalis*, *E. faecium*, *E. gallinarum*, *Enterocloster lavaliensis*, *E. clostridioforme*, *Angelakisella massiliensis*).

### 2.5. vanY

The protein vanY was found to be linked to the vanA and vanM operons only; two main groups were identified (Figure S5). The first contained mostly *Bacillus*, *Neobacillus*, *Peribacillus*, *Paenibacillus* and *Clostridium* species. Within the second group, in the basal position, a grade-like structure could be identified, with short internal branch lengths containing different clusters of Bacillus and related species, also including a cluster containing vanM-producing *E. faecium* isolates (vanRSMHY on a plasmid), *Aerococcus urinaeequi*, *Klebsiella variicola* and *K. pneumoniae*, together with different *Bacillus* spp. The next branching separated a single sequence of *Neobacillus sedimentimangrowi*, followed by the separation of a group of *Bacillus thuringiensis* strains. A group of *Virgibacillus*, *Neobacillus*, *Brevibacillus*, *Bacillus* and *Paenibacillus* species appeared to be the sister lineage of several closely related proteins from vanA-producing organisms.

### 2.6. vanZ

The protein vanZ occurred only in association with the vanA operon. The basal group contained *Tumebacillus* and *Rummeliibacillus* spp., without any glycopeptide resistance ligase proteins (Figure S6). The stem was a poorly resolved group of proteins from different Bacillales containing a distinguishable clade of *Desulfosporosinus* and *Clostridium* spp. in which glycopeptide resistance ligases were not found; the crown group corresponded to the crown group of the vanA tree.

### 2.7. vanR

The sequences of vanR associated with different D-Ala-D-Ala ligases of vancomycin-resistant enterococci showed major differences; enterococcal vanR sequences associated with a certain ligase were never recovered when vanR associated with another ligase type was used as the query using the conditions shown in the methods.

#### 2.7.1. vanR-A

**Figure 2:**
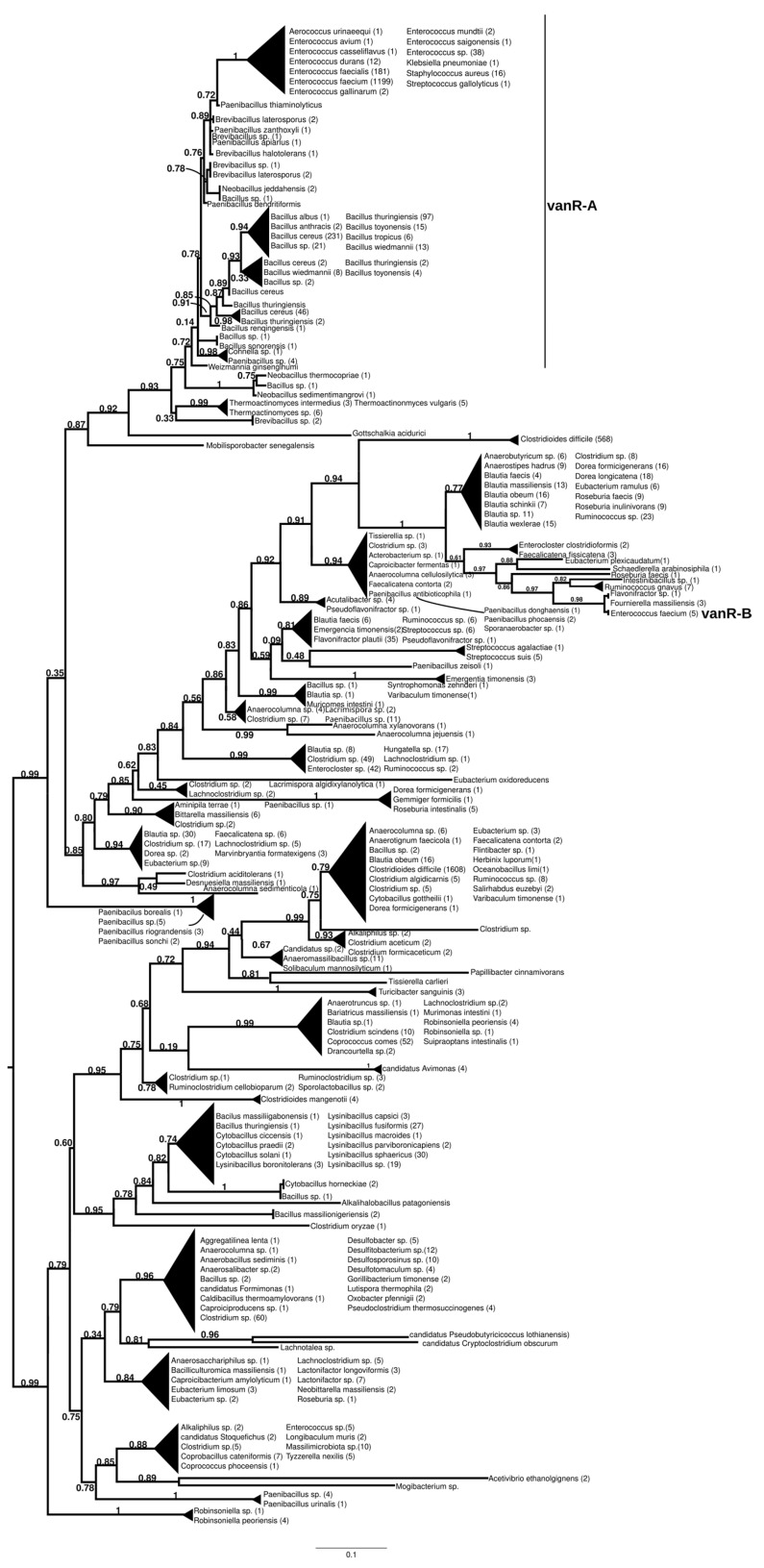
Phylogenetic tree of D-Ala-D-lactate ligases as reconstructed based on a vanR-A (GenBank Accession number: KR047792) query after filtering Blast hits for 60% aa identity and ≥90% coverage. Clades were collapsed to reduce redundancy on the tree. Root is placed based on the result of MAD 2.2. Support values refer to the SH-like local support calculated by FastTree. For the full phylogenetic trees and MAD root placement probabilities see Supplementary Materials (Figure S7).

The homologs of the vanR proteins from the vanA producers formed two well-supported major clusters (Figure 2 and Figure S7). One was a large cluster, in which the most basal group of proteins was derived from different *Paenibacillus* spp., including *P. jilunlii* and *P. sonchii*, with ligases basal to vanA. Towards the tip of the phylogenetic tree, two main groups appeared with low support values, for one of which proteins from *Clostridium aciditolerans*, *Desnuesiella massiliensis* and *Anaerocolumna sedimenticola* were the first branch, followed by a complex group of sequences from various members of Clostridia and Actinobacteria. This large cluster also included vanR sequences from five vanB carrier *E. faecium*, whose vanRs are not linked to the vanB operon, as well as a highly homogeneous cluster from *Clostridioides difficile* (with a chromosomal vanRSG#T, where # is a metallopeptidase corresponding to vanX, vanY or vanXY). Furthermore, it included a cluster of sequences derived from vancomycin-susceptible *Streptococcus agalactiae* (vanRS in multiple copies, one of them associated with a Dlt operon coding for D-alanyl-(lipo)teichoic acid biosynthesis, another with a metallopeptidase) and *S. suis* (multiple vanRS types without resistance ligases). The earliest branches of the other group were proteins from *Gottschalkia acidurici* and *Mobilisporobacter senegalensis*, followed by protein sequences from *Brevibacillus* spp. and *Paenibacillus* spp., which also included a large clade of closely related proteins from *Bacillus cereus* s. l. The vanA-associated vanR proteins from *E. faecium*, *E. faecalis*, *E. saigonensis*, *E. mundtii*, *E. durans*, *E. casseliflavus*, *E. avium*, *Aerococcus urinaeequi*, *S. aureus*, *S. gallolyticus* and *Pseudomonas stutzeri* appeared to be the crown group of this cluster.

The sister group to these was a second cluster with proteins from *Robinsoniella* (harboring multiple vanRS copies, including a vanRSZ gene cluster and multiple copies of vanRS upstream of the ABC transporters) in the stem. Further basal branching was poorly resolved, containing proteins from *Paenibacillus*, *Lachnoclostridium*, *Lactonifractor*, *Lachnotalea* and *Eubacterium*, but three well-defined sister groups were able to be identified. One of the three clusters contained proteins from *Clostridioides mangenotii* as the most basal branch, with several generally well-resolved clusters with proteins from various Clostridia and Bacillaceae in the stem, also including vanR from the same *Robinsoniella* genomes but only distantly related to the stem vanRs from *Robinsoniella*. Further branches were proteins from various species belonging to Clostridia (*Ruminococcus*, *Blautia*, *Dorea*, *Anaerotignum*, *Pseudoruminococcus*, *Roseburia*, *Faecalicatena*), including another large cluster of proteins from *C. difficile* (vanRSGYT). The second of the three clusters was a well-defined clade with vanR from *Clostridium oryzae* as the most basal, with vanRs from *Cytobacillus*, *Alkalihalobacillus* and *Bacillus* spp. as well as from *Clostridium* spp. in the stem and with a number of sequences from *Lysinibacillus*. The third cluster comprised two sister groups, one with *Tissierella*, *Clostridium* and *Tyzzerella* proteins in the stem and a few sequences from *Enterococcus* spp. (with vanRS in multiple copies, one of them next to an ABC transporter gene; vanRS upstream to the D-Ala-D-Ala carboxypetidase and ligase gene cluster; vanR alone close to a D-Ala-D-Ala ligase, carboxypeptidase and serine racemase) and *E. florum* (vanRYS). The sister to this group contained proteins from different Clostridia (*Clostridium*, *Pseudoclostridium*, *Desulfosporosinus*, etc.).

#### 2.7.2. vanR-M

**Figure 3:**
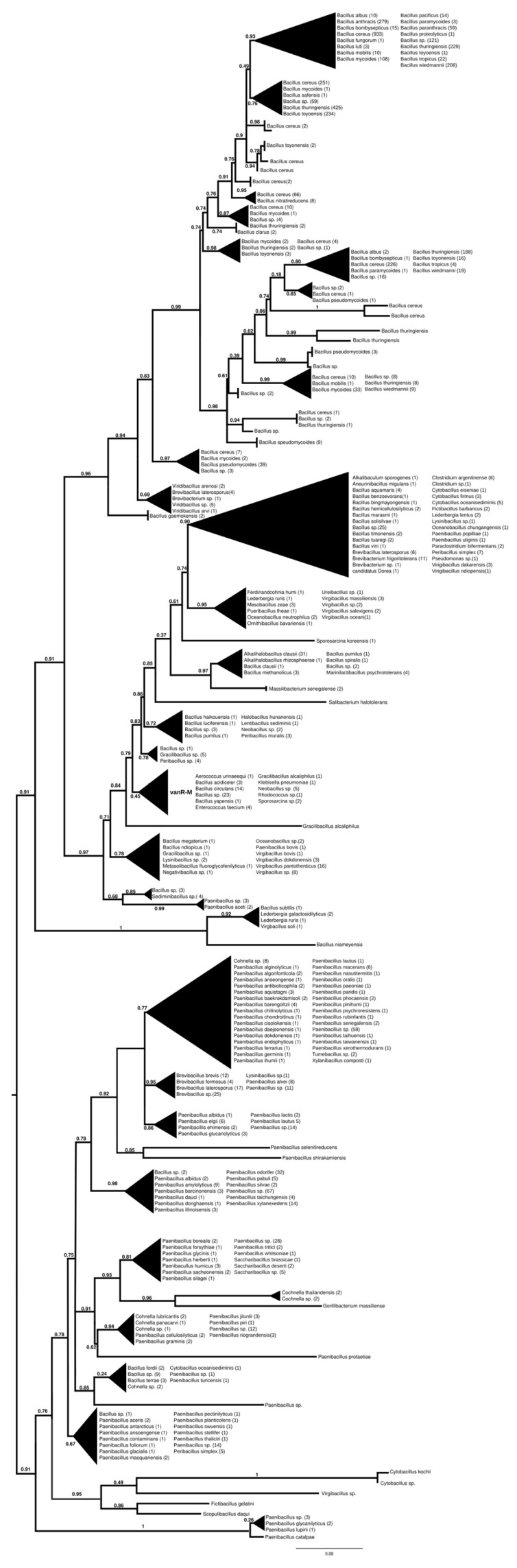
Phylogenetic tree of D-Ala-D-lactate ligases as reconstructed based on a vanR-M (GenBank accession number: FJ349556) query after filtering BLAST hits for 60% aa identity and ≥90% coverage. Clades were collapsed to reduce redundancy in the tree. Roots are placed based on the result of MAD 2.2. Support values refer to the SH-like local support calculated using FastTree. For the full phylogenetic trees and MAD root placement probabilities, see Supplementary Materials (Figure S8).

The proteins found using vanR associated with vanM as a query were found in various members of Bacillales, frequently in *Paenibacillus* spp., which was also the most basal branch (Figure 3 and Figure S8). The next branch contained a wealth of response regulators of the *Bacillus cereus* complex, followed by the separation of two sister groups, one containing vanRs from various Bacillales, including vanR from *Paenibacillus popilliae,* harboring vanF, and the other containing regulators of *Bacillus circulans* and other *Bacillus* spp. in the stem, as well as a small monophyletic clade of enterococcal vanR-Ms, formed together with one vanR-M from *Aerococcus urinaeequi* and one from *Klebsiella pneumoniae*.

#### 2.7.3. vanR-B

**Figure 4:**
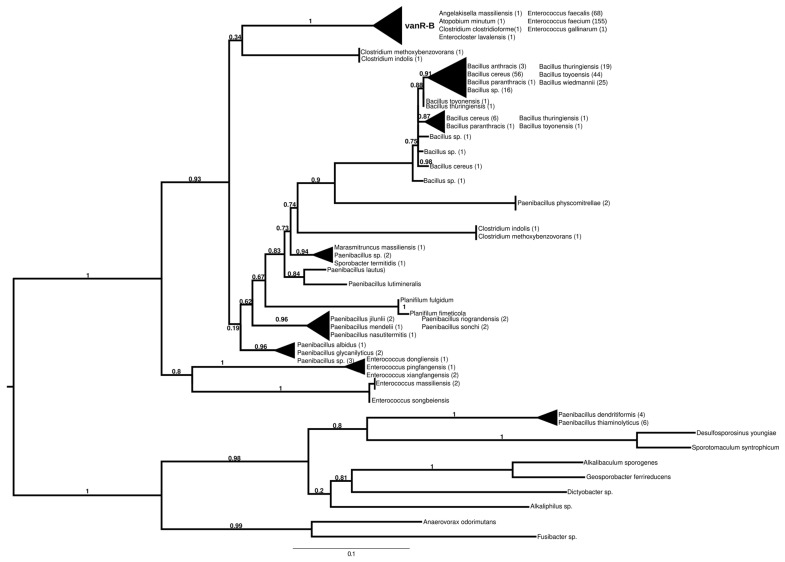
Phylogenetic tree of D-Ala-D-lactate ligases as reconstructed based on a vanR-B (GenBank Accession number: KC489787) query after filtering BLAST hits for 60% aa identity and ≥90% coverage. Clades were collapsed to reduce redundancy in the tree. Roots are placed based on the result of MAD 2.2. Support values refer to the SH-like local support calculated using FastTree. For the full phylogenetic trees and MAD root placement probabilities, see Supplementary Materials (Figure S9).

The proteins found using vanR associated with vanB as a query formed two major groups (Figure 4 and Figure S9). In the most basal branch of the first group clustered vanRs from the food-derived enterococci (e.g., *E. pingfangensis*) harboring ligases clustering with vanF proteins (Figure 1 and Figure S1). Two sister groups were then found, one of which contained *Clostridium indolis* and *C. methoxybenzovorans* in the stem and vanRs from vanB producers (*E. faecium*, *E. faecalis*, *E. gallinarum*, *Atopobium minutum*, *Enterocloster lavalensis*, *Clostridium clostridioforme* and *Angelakisella massiliensis*), largely mirroring the branching of the vanB proteins (Figure 1 and Figure S1). The other group contained vanRs from different *Paenibacillus* spp. in its stem, including *P. jilunlii* and *P. sonchii*, which harbored a ligase basal to the vanA clade (Figure 1 and Figure S1); in the next branch were other vanRs from *Clostridium indolis* and *C. methoxybenzovorans* different from those in the formerly mentioned group and then vanRs from *P. physcomitrellae*, with a ligase clustering in the stem of the group harboring vanF (Figure 1 and Figure S1) and proteins from *Bacillus cereus sensu lato* as the crown group. The other major group comprised proteins from *P. thiaminolyticus* and *P. dendritiformis* and various soil and ocean bacteria with variable van genes.

#### 2.7.4. vanR-D

**Figure 5:**
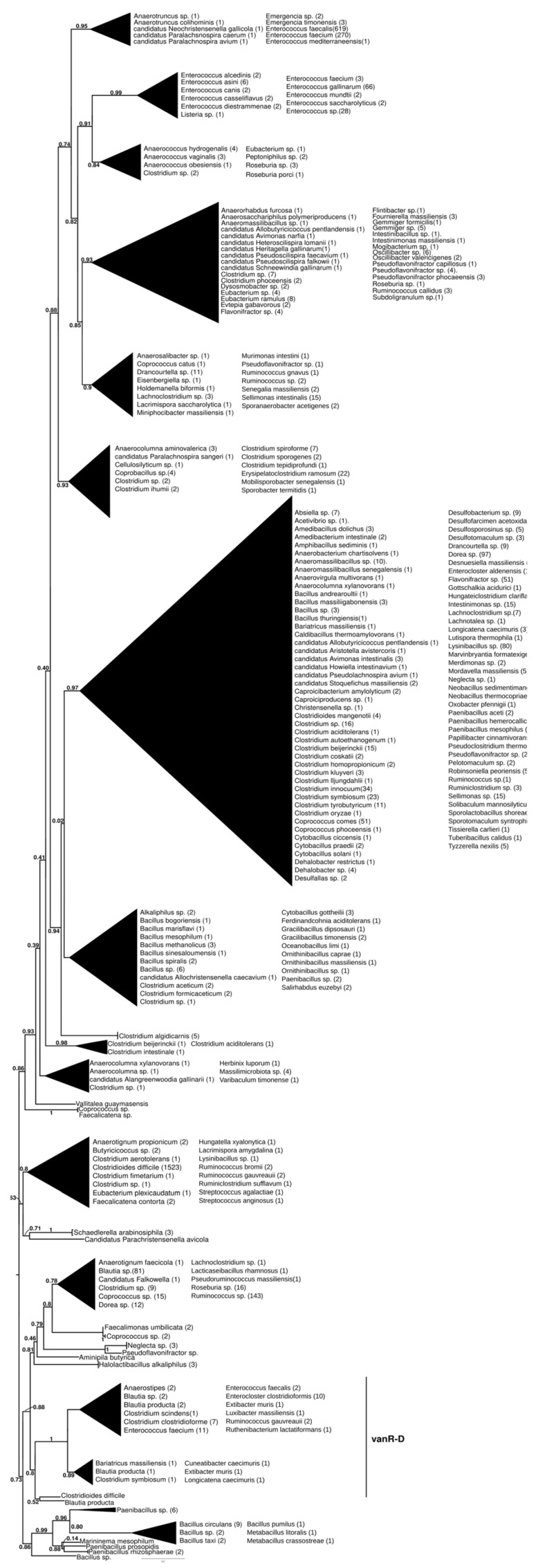
Phylogenetic tree of D-Ala-D-lactate ligases as reconstructed based on a vanR-D (GenBank Accession number: AF175293) query after filtering BLAST hits for 60% aa identity and ≥90% coverage. Clades were collapsed to reduce redundancy in the tree. Roots are placed based on the result of MAD 2.2. Support values refer to the SH-like local support calculated using FastTree. For the full phylogenetic trees and MAD root placement probabilities, see Supplementary Materials (Figure S10).

The basal branching of putative sister groups of vanR-D was not unequivocal; these included a *Paenibacillus*-derived protein group; a group of vanRs from *Clostridioides difficile*, *Streptococcus anginosus*, *S. agalactiae* and various Lachnospiraceae and a monophyletic clade containing vanRs from many Clostridia and Bacillales (Figure 5 and Figure S10). The latter group included two major clades, one containing vanRs from animal-derived enterococci (*E. casselliflavus*, *E. gallinarum*, *E. asini*, *E. diestrammenae*, *E. alcedinis* and *E. canis*), together with vanRs from a vanA-producing *E. faecium*, an *E. faecium* without a resistance ligase, a vanN-producing *Enterococcus* sp. and a vanC-producing *Listeria* isolate, while the other contained two monophyletic sister clades of vanRs from *E. faecium* with variable resistance patterns (vanA, vanM or without glycopeptide resistance) and vanRs from glycopeptide-susceptible *E. faecalis* isolates. The vanR protein clusters from different *Enterococcus* species strikingly coincided with species identity in these latter groups.

In another early diverging branch, the vanR proteins associated with vanD-producing enterococci formed a clade closely corresponding to and including most of the taxa of the vanD protein phylogeny but not the vanRs of *C. indolis* and *C. methoxybenzovorans*, located basally in the vanD phylogeny (Figure 1 and Figure S1). 

#### 2.7.5. vanR-F

When vanR of the vanF-producing *Paenibacillus popilliae* was used as a query, the dendrogram yielded was very close to that produced using vanR-M (Figure 3 and Figure S8).

### 2.8. vanS

Similarly to vanR, proteins from enterococci harboring different ligases showed major sequence differences, and generally, enterococcal vanS proteins associated with a certain ligase were rarely found when vanS proteins associated with other ligases were used in the queries. Generally, the topologies of the phylogenies were highly similar to those found for the different vanRs. 

#### 2.8.1. vanS-A

The basal protein in this phylogeny was from *Clostridium aciditolerans*, which only contained a vanRS gene pair without a resistance ligase (Figure S11). The earliest diverging branches included proteins from *Neobacillus* (*N. sedimentimagrovi* with vanRSY; *N*. *thermocopriae* with vanRSZ), *Paenibacillus* (vanXS) and some Clostridia-related bacteria (*Anaerocolumna aminovalerica*, *Oscillibacter valericigenes* and *Desnuesiella massiliensis*, all containing a vanG operon) and a closely related cluster of *Thermoactinomyces* spp. (vanHAXRSZ), as well as a few genomes from *Brevibacillus* (vanRSHAXY). The next branch comprised two sister groups, one of which was a large cluster of proteins from *Bacillus* spp., with proteins from *Paenibacillus thiaminolyticus* and *P. dendritiformis* (vanRSY, also containing vanGXT without vanRS), *P. apiarius* (vanRSHAXYZ), *P. piri* (vanRSWHAX), *Alicyclobacillus shizuokensis* (RSHAXY), *Neobacillus yeddahensis* (vanRSHAXZ) and *Neobacillus* sp. (vanYS) in the stem, corresponding to the branching structure of vanA (Figure 1 and Figure S1), together with proteins from *B. cereus s.l.*, in which vanRS is frequently associated with vanY (these do not have D-Ala-D-Ala ligases). The other sister group contained vanS from *Weizmannia ginsengihumi* (vanRS, vanY, vanZ distantly positioned in the genome) in the stem and a uniform cluster of vanA-associated vanS proteins from enterococci; *E. faecium* (vanRSYHM on a plasmid, vanRS) and *E. faecalis* (vanRS without resistance ligase) were not separated. This group also included proteins from other *Enterococcus* species, *E. durans* (vanRS, vanRSHAXYZ), *E. mundtii* (vanRS, vanRSHAX), *E. avium* (vanRS), *E. gallinarum* (vanCXTRS), *E. casseliflavus* (vanCXTRS), *Enterococcus* spp. (animacy, vanSR; vanHAX, vanYZ, vanSR separately), *E. saigonensis* (on the chromosome, only vanS; on a plasmid, vanRSHAXYZ), *Streptococcus gallolyticus* (vanSRHAX), *Aerococcus urinaeequi* (vanRSZ) and several *Staphylococcus aureus* genomes (vanRSHAXYZ).

#### 2.8.2. vanS-M

This phylogenetic tree (Figure S12) was practically identical to that for vanR-M (see Figure 4).

#### 2.8.3. vanS-B

In the case of vanS-B, the basal group comprised proteins from *Paenibacillus* spp. with vanG ligases (Figure S13). The next branch was two sister groups, one of which showed branching similar to that exhibited by vanB (see Figure 1 and Figure S1). The sister to this was a group of proteins from different bacilli, where the stem included *Marasmitruncus massiliensis*, different *Paenibacillus* spp. without resistance ligases (vanRSZ or vanRSY), *Clostridium methoxybenzovorans* and *Lacrimispora indolis* harboring vanD-related ligases and *Paenibacillus physcomitrellae* harboring vanF-related ligases, while the more derived proteins were from *Bacillus cereus s.l.* without resistance ligases, where vanS was associated with vanY or vanZ. 

#### 2.8.4. vanS-D

This dendrogram was identical to the part of the vanR-D dendrogram (Figure 5 and Figure S10) including the vanR-Ds of vanD-producing enterococci and related vanRs (Figure S14). This was also very similar to the corresponding branch on the vanX tree (Figure S3).

#### 2.8.5. vanS-F

When vanS of the vanF-producing *Paenibacillus popilliae* was used as a query, the dendrogram yielded was identical to that produced using vanS-M (Figure S12).

#### 2.8.6. Structural Evolution of the D-Ala-D-lactate Synthesizing Glycopeptide Resistance Operons

**Figure 6:**
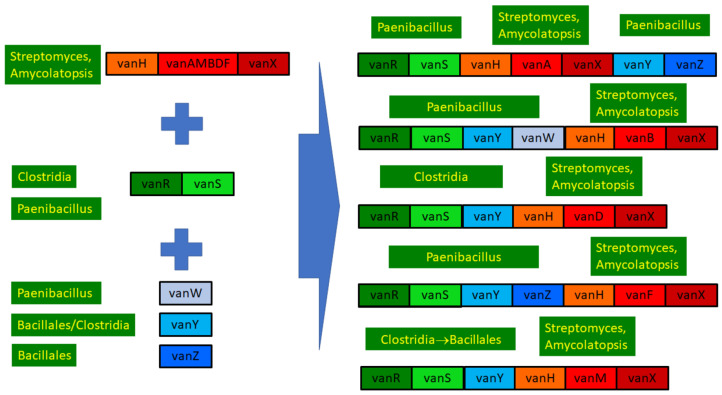
Overview of the structure and evolution of D-ala-D-lactate synthesizing glycopeptide resistance operons.

Phylogenetic results were used to review the structure and evolution of D-Ala-D-lactate synthesizing glycopeptide resistance operons (Figure 6).

## 3. Discussion

Published analyses on the phylogeny of the D-Ala-D-lactate ligases associated with glycopeptide resistance agree in terms of the relatedness of vanA, vanM, vanB, vanD and vanF [4,6,7], while D-Ala-D-lactate ligases of lactic acid bacteria conferring generic glycopeptide resistance have been recognized as unrelated [4,8,9,10,11]. Accordingly, in the present study, ligases from lactic acid bacteria were never encountered with the similarity threshold set at 60%. However, the relationships among vanA, vanM, vanB, vanD and vanF are unresolved; the close relationship between vanA and vanM is unequivocal, but reports are contradictory regarding the phylogenetic positions of vanB, vanD and vanF [6,7,12]. In this study, the amino-acid-level phylogeny of the complete ligase proteins showed that vanB and vanD are sister groups, as confirmed by their shared characteristics of not conferring resistance to teicoplanin [13]. The clade vanM-vanA is a sister to vanB-vanD, while the putative basal position of vanF originating from *Paenibacillus popilliae* [14] is controversial. Except for vanF, all the types occurred in enterococci, primarily in *E. faecium* and *E. faecalis*, and were relatively conserved, while vanF was found only in nonpathogenic enterococci (e.g., *E. pingfangensis*) and showed a more pronounced sequence variability. The clustering of the newly reported vanP [15] among the vanM protein sequences suggests that vanP may be a variant of vanM. Practically, only vanA and vanB occurred in human pathogenic Gram-positive bacteria other than enterococci, primarily in *Staphylococcus aureus* resistant to methicillin (MRSA). The close relatedness of these to enterococcal vanA/vanB implies that enterococci served as the source of these genes and proteins for other human pathogens [16,17].

Both the vanA and vanM clusters were characterized by sequences from spore-forming bacteria in the stems, suggesting that these, especially the genera *Paenibacillus* and *Brevibacillus*, served as a direct source for the enterococcal vanA and vanM genes [18]. The ligase genes may have evolved in these bacilli into vanA, vanM and vanF, while the vanB-vanD clade may have evolved in other bacteria, in enterococci or in yet unknown species, as suggested by the absence of stem taxa for vanB. However, this may derive from the main limitation of the study, which is that the phylogenies are based on genomes from the reference genome database; other published non-reference genomes or upcoming new genomes may refine the phylogenetic relationships inferred or clarify unequivocal issues.

The ligase vanD may have evolved from vanB or vanB ancestors in bacteria associated with the human or animal gut microbiome, such as *Ruminococcus*, *Blautia* and/or *Clostridium* [19]; horizontal transfer of vanD to enterococci, in contrast to the cases of other ligases, probably took place multiple times.

Soil-dwelling spore-forming bacilli, primarily *Paenibacillus* spp., appear in the stem groups frequently, confirming that the genus *Paenibacillus* plays a key role as a source of vancomycin resistance ligase genes for enterococci [18,20,21,22,23], which is in line with their suggested central role in resistance spread networks in environmental microbial ecosystems [24]. However, the genus *Paenibacillus* is unlikely to be the ultimate source, as many strains are susceptible to glycopeptides [21,22,25,26], and the resistomes are not structured by phylogeny [27]. Thus, as suggested earlier [21,22,25,26], (paeni)bacilli acted as transfer organisms in the transfer of ligase genes to the clinically relevant enterococci from glycopeptide-producing *Streptomyces*/*Amycolatopsis* species, which form a large sister clade of ligase proteins, indicating that they are the ultimate and very ancient source of these genes for enterococci and (paeni)bacilli [28,29], as well as for other soil-dwelling organisms that do not produce glycopeptides [6,30]. Analysis of the split fragments indicates that vanA and vanB were transferred as whole genes and are unlikely to be the result of recombination.

Furthermore, the proteins most basal to this large clade containing all groups of human importance are associated with bacteria of land plants (*Rathayibacter* spp. and *Phytohabitans suffuscus*), which points out the importance of the soil as a potential site for the horizontal transfer of these genes to enterococci, also found in soils or associated with plants, including food cropa [31,32,33,34]. In turn, such plant-associated enterococci, including *E. faecalis* and *E. faecium*, may easily reach the human gut directly through plant-based food or through production animals [35,36,37].

Similarities in the backbone structure of the phylogeny of the ligases and the associated vanH (lactate dehydrogenase) and vanX (dipeptidase) proteins suggest that the *Streptomyces*/*Amycolatopsis* clade also serves as an ultimate source for the other core genes of the van operons as well. The operon vanA was most probably transferred as a whole operon (vanHAX), but important controversies were revealed for the other ligase types. In the case of the dehydrogenases (vanH), those associated with vanA (vanH-A) were distant from vanH-M, which was a sister to vanH-F, forming an earlier diverged group. In the case of the dipeptidases (vanX), the basal protein sequences belong to vanX-D, while vanX-F is close to vanX-B. The nonpathogenic enterococci harboring a vanF homolog did not possess vanH or vanX.

In contrast, homologs of the accessory resistance genes vanZ, vanY and vanW or the regulatory proteins vanS and vanR queried were never found in *Streptomyces* or related bacteria, in line with the major differences between the GC proportions in core and accessory genes [22]. All three accessory protein phylogenies contained sequences from various soil- or plant-associated spore-forming rods in their stems; thus, the genes vanW, vanY and vanZ probably originate from bacteria of the order Bacillales [18]. The gene vanW was transferred to enterococci carrying vanB [5], and basal vanW carriers harbored various ligases, some of which clustered with vanA, some of which clustered with the vanB-vanD clade and another which clustered with vanM, while the most basal harbored a ligase clustering close to the ligases from the glycopeptide producers. The gene vanY was acquired at least twice independently, by enterococci with vanA and by those with vanM; probably, the same ancestral vanY genes were the source of vanY associated with vanA and vanM, suggesting that linking of the accessory vanY gene occurred prior to the transfer of the operon to enterococci, the probable source being a species of the Bacillaceae family. The vanZ protein was exclusively associated with vanA-producing enterococci; the probable source was again a member of Bacillaceae. These assumptions are substantiated by the presence of vanY in the vanZYHFX operon of vancomycin-resistant *Paenibacillus popilliae*, as well as in the closely related *P. lentimorbus*, which is vancomycin-susceptible and produces no resistance ligases [38].

As expected, the vanR-vanS protein pair yielded very similar phylogenies. Generally, phylogenetically distinct vanRS copies were associated with vanA, vanM, vanB and vanD, while vanF-associated vanRS was related to vanRS associated with vanM, which is in line with the different mechanisms of sensing and regulation between different vanRS copies [39]. None of these vanRS copies were related to the homologs from the glycopeptide producers, as expected based on their GC proportion differences [22]; thus, the types of vanRS regulating the different operons were probably acquired from different members of Clostridia and Bacillaceae (Figure 6). The transfer may have taken place together with the accessory genes vanZ or vanY, as closely related vanR and vanS types were found both in bacteria with and without the ligase, linked to vanZ and/or vanY in the latter (including, e.g., *Enterococcus florum* in this study). Strikingly, the vanF operon of the abovementioned vancomycin-resistant *Paenibacillus popilliae* was associated with a vanRS, which was closest to the vanRS of *P. lentimorbus* (vancomycin-susceptible without the ligase), which also bears a vanY gene [38].

Acquisition of the vanRS gene pair by enterococci unrelated to the resistance operon was also detected, as indicated by the clustering of vanRS-D related proteins according to species. However, inconsistency with the enterococcal phylogenies based on 16S rDNA or the core genome suggests that this may have spread through horizontal transfer between *Enterococcus* species [40,41]. The similarities in the topology within the monophyletic clades vanB, vanR-B and vanS-B indicate that vanRS acquisition predated vanB splits and may have been acquired by ancestral vanB-carrying isolates. 

In line with their ubiquitous nature [39], multiple copies of vanRS pairs, which are distantly related, are present in the genomes; sometimes, the vanRS regulating a glycopeptide resistance operon in an isolate may regulate other genes in others [38], as shown in this study by the vanR-A in the vanB-producing enterococci not being associated with the vanB operon.

In conclusion, the ultimate source of all the core glycopeptide resistance operons was most probably among the glycopeptide-producing streptomycetes. The accessory genes vanW, vanY and vanZ were conjoined in various *Bacillus*/*Paenibacillus* species through multiple gene acquisition events. The regulatory genes were probably also acquired multiple times by enterococci from (paeni)bacilli, in which vanRS gene pairs are ubiquitously present, frequently associated with accessory genes in glycopeptide-susceptible species but not with resistance ligases.

## 4. Materials and Methods

The amino acid sequences of the genes located in the vancomycin resistance gene cluster of *Enterococcus faecium* (vanA: KR047792 and vanB: KC489787) were used as a query to search against a custom BLAST [42] database. After initial screening of the sequence variability, the vanR and vanS genes were found to show a higher sequence variability relative to the other members of the gene cluster (see Results). To reconstruct the detailed phylogenetic relationships of these genes, vanR and vanS amino acid sequences of *Paenibacillus popilliae* (NZ_BALG01000075.1) containing vanF and three other enterococci (KC489787, AF175293 and FJ349556, harboring vanB, vanD and vanM, respectively) were also used as search queries. We downloaded the aa sequences of all the bacterial genomes (n = 219,549) listed in the NCBI RefSeq database (downloaded 2 July 2021). Genomes obtained in FASTA format were edited to include the species name and accession number in the identifier of each sequence for easier downstream processing. Then, makeblastdb 2.10.1+ was used to format the sequence database, which was searched using blastp 2.10.1+. The highest scoring pairs in the custom database for each gene in the gene cluster were screened in independent analyses. BLAST hits with at least 60% aa identity and at least 90% query coverage were used for subsequent analyses. Sequences were aligned using MAFFT 7.471 [43]; then, the phylogenetic trees were reconstructed using FastTree 2.1.10 [44] with the default parameters, and statistical robustness was assessed according to the SH-like local support values. The phylogenetic trees were rooted using the minimal ancestor deviation method, as implemented in MAD 2.2 [45]. The resulting phylogenetic trees were visualized in FigTree 1.4.4 (http://tree.bio.ed.ac.uk/software/figtree/) and further edited in Inkscape 0.92.4.

Reference sequences for vanA (ADM24920) and vanB (AHH83931) were split into 7 fragments of 50 amino acids and were used as queries for BLASTp analysis against the NCBI non-redundant database using the -remote option. The 100 best homologous sequences with aa identity ≥60% and coverage ≥90% were selected for further analysis. From these sequences, scientific names were collected and counted to represent the distribution of hits for each fragment.

## Data Availability

This work was presented in part at the 32nd European Congress of Clinical Microbiology and Infectious Diseases, Lisbon, Portugal. All the phylogenies are provided as Supplementary Materials. Further inquiries are welcomed by the authors.

## Supplementary Materials

The following supporting information can be downloaded at https://www.mdpi.com/article/10.3390/antibiotics13070573/s1. Figure S1: Extended tree of vanAB; Figure S2: Extended tree of vanH; Figure S3: Extended tree of vanX; Figure S4: Extended tree of vanW; Figure S5: Extended tree of vanY; Figure S6: Extended tree of vanZ; Figure S7: Extended tree of vanR-A; Figure S8: Extended tree of vanR-M; Figure S9: Extended tree of vanR-B; Figure S10: Extended tree of vanR-D; Figure S11: Extended tree of vanS-A; Figure S12: Extended tree of vanS-M; Figure S13: Extended tree of vanS-B; Figure S14: Extended tree of vanS-D.
