# Supplementary figures and images for: Phylogenetic Analysis of the Genes in D-Ala-D-Lactate Synthesizing Glycopeptide Resistance Operons: The Different Origins of Functional and Regulatory Genes
Source: Antibiotics (Basel). 2024 Jun 21;13(7):573. doi: 10.3390/antibiotics13070573 (PMC11273654; doi:10.3390/antibiotics13070573)

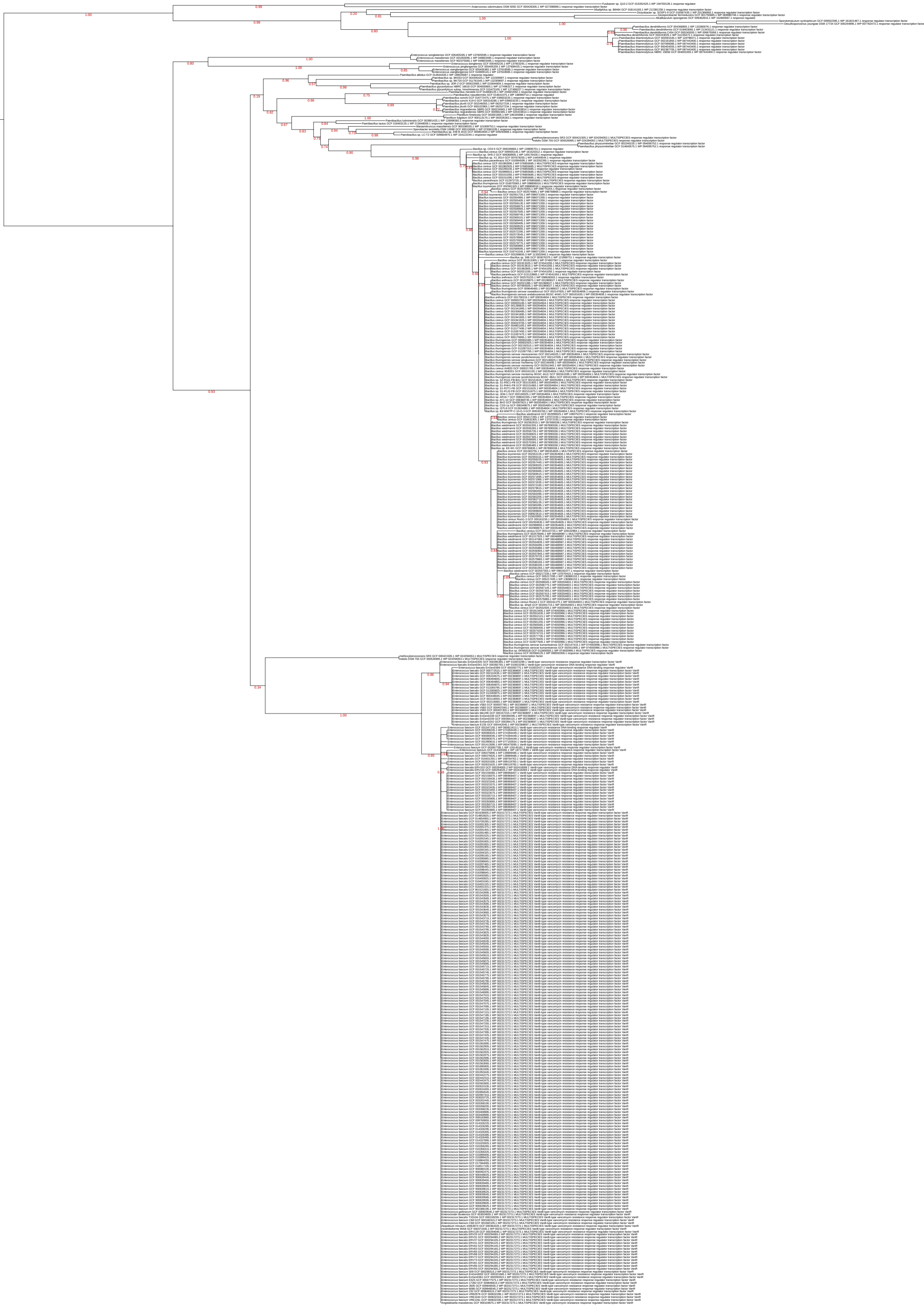

Supplement: Supplementary file 1 [file antibiotics-13-00573-s001.zip › FigureS9_Extended tree of vanR-B.pdf]

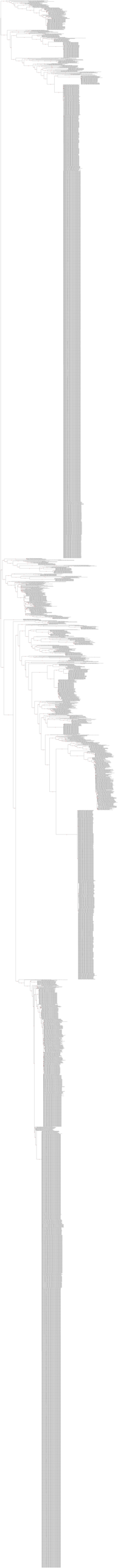

Supplement: Supplementary file 1 [file antibiotics-13-00573-s001.zip › FigureS7_Extended tree of vanR-A.pdf]

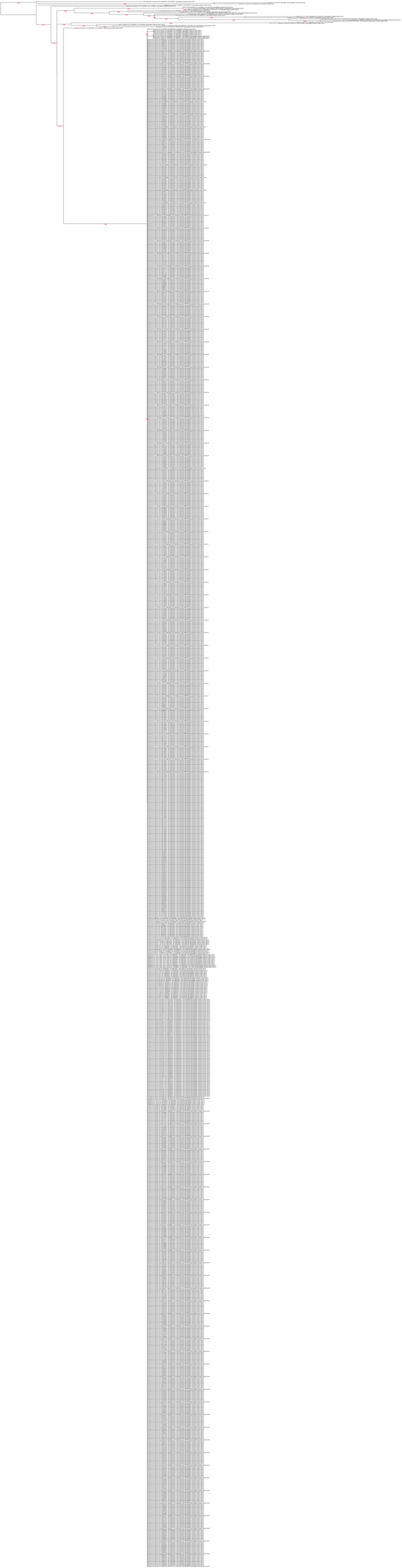

Supplement: Supplementary file 1 [file antibiotics-13-00573-s001.zip › FigureS6_Extended tree of vanZ.pdf]

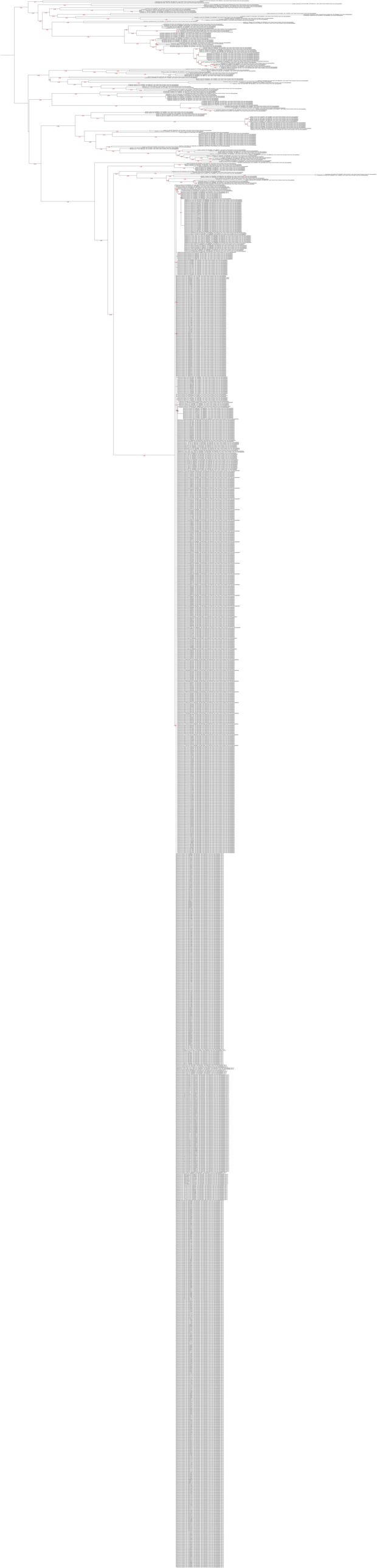

Supplement: Supplementary file 1 [file antibiotics-13-00573-s001.zip › FigureS5_Extended tree of vanY.pdf]

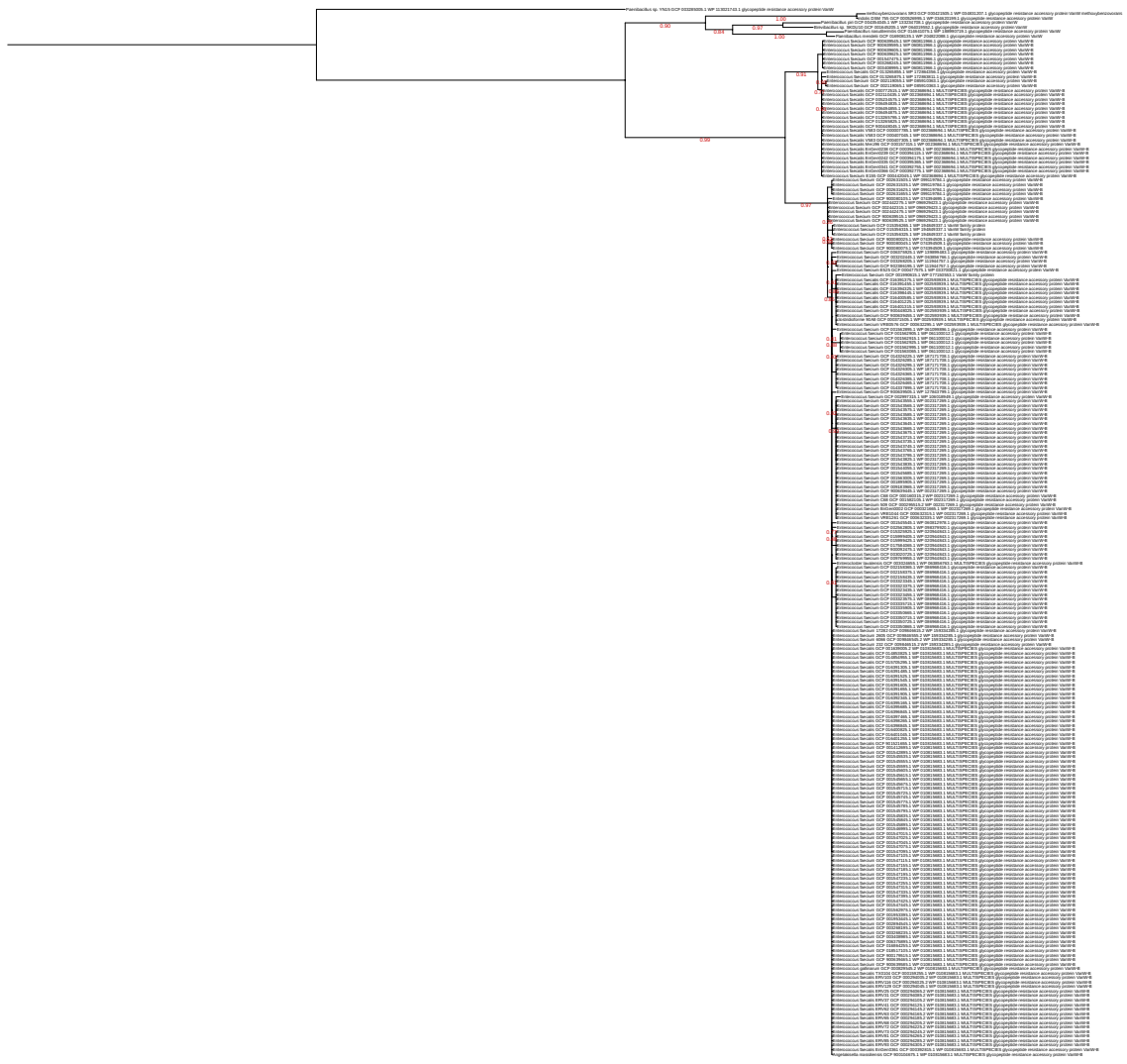

Supplement: Supplementary file 1 [file antibiotics-13-00573-s001.zip › FigureS4_Extended tree of vanW.pdf]

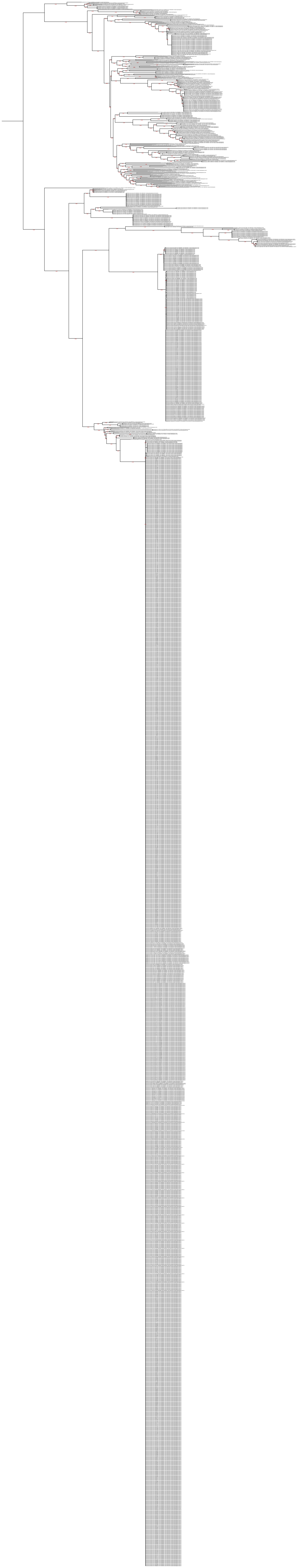

Supplement: Supplementary file 1 [file antibiotics-13-00573-s001.zip › FigureS2_Extended tree of vanH.pdf]

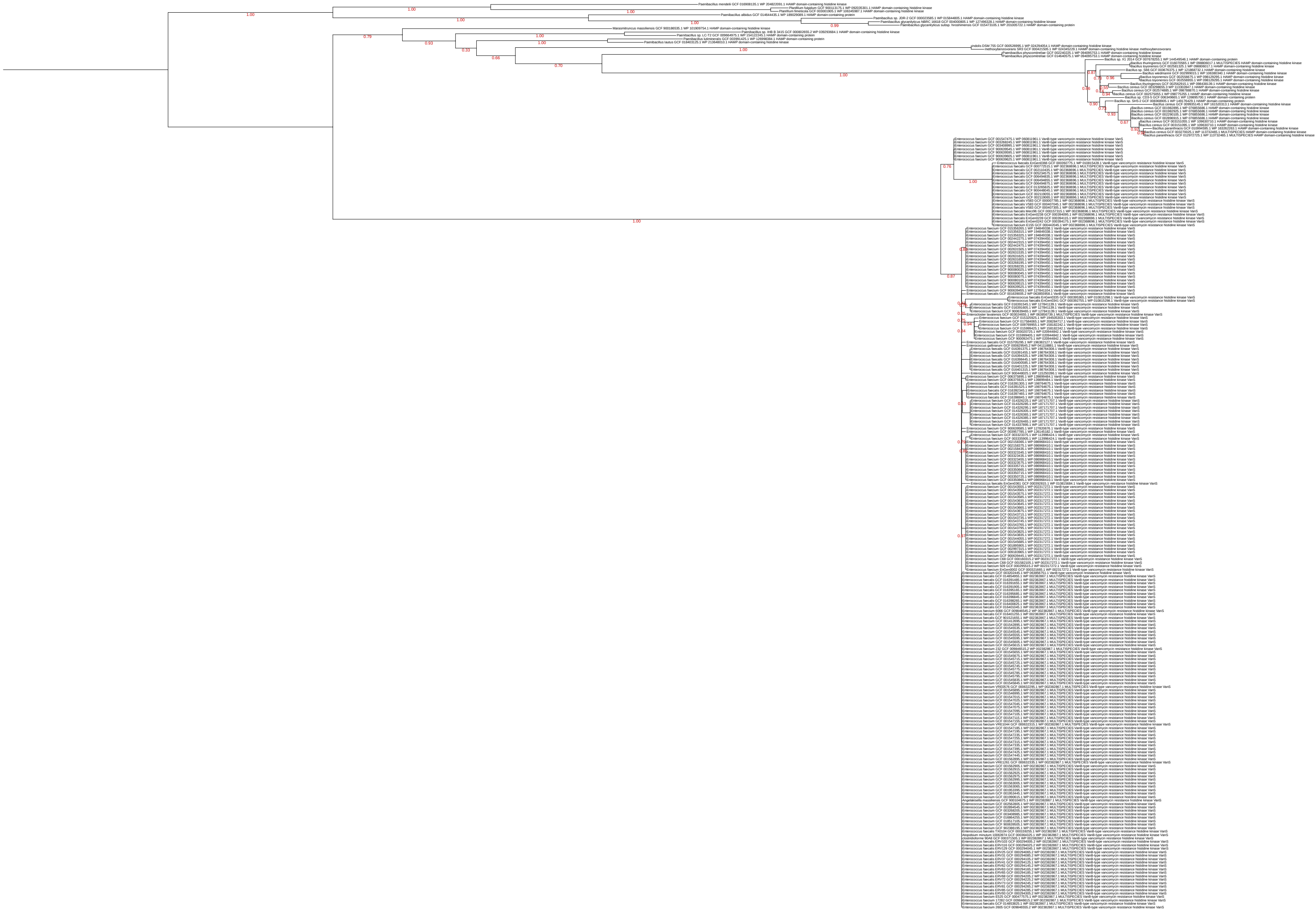

Supplement: Supplementary file 1 [file antibiotics-13-00573-s001.zip › FigureS13_Extended tree of vanS-B.pdf]

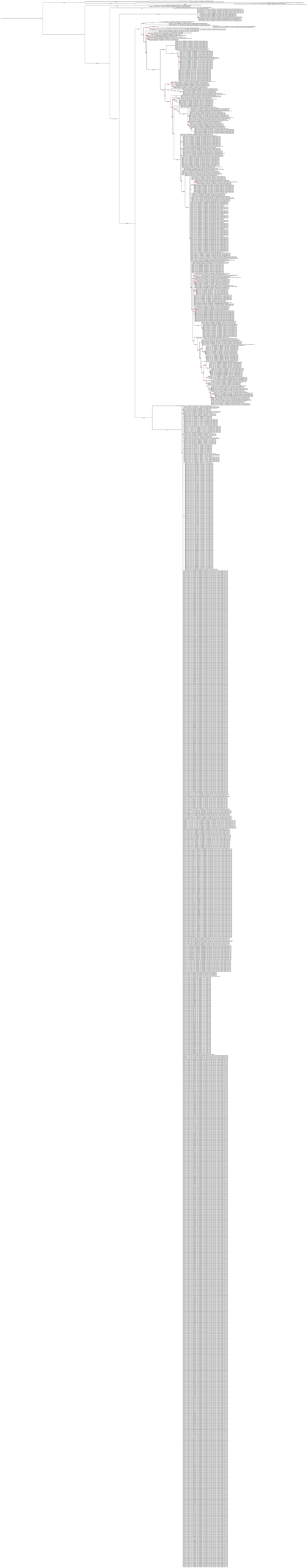

Supplement: Supplementary file 1 [file antibiotics-13-00573-s001.zip › FigureS11_Extended tree of vanS-A.pdf]

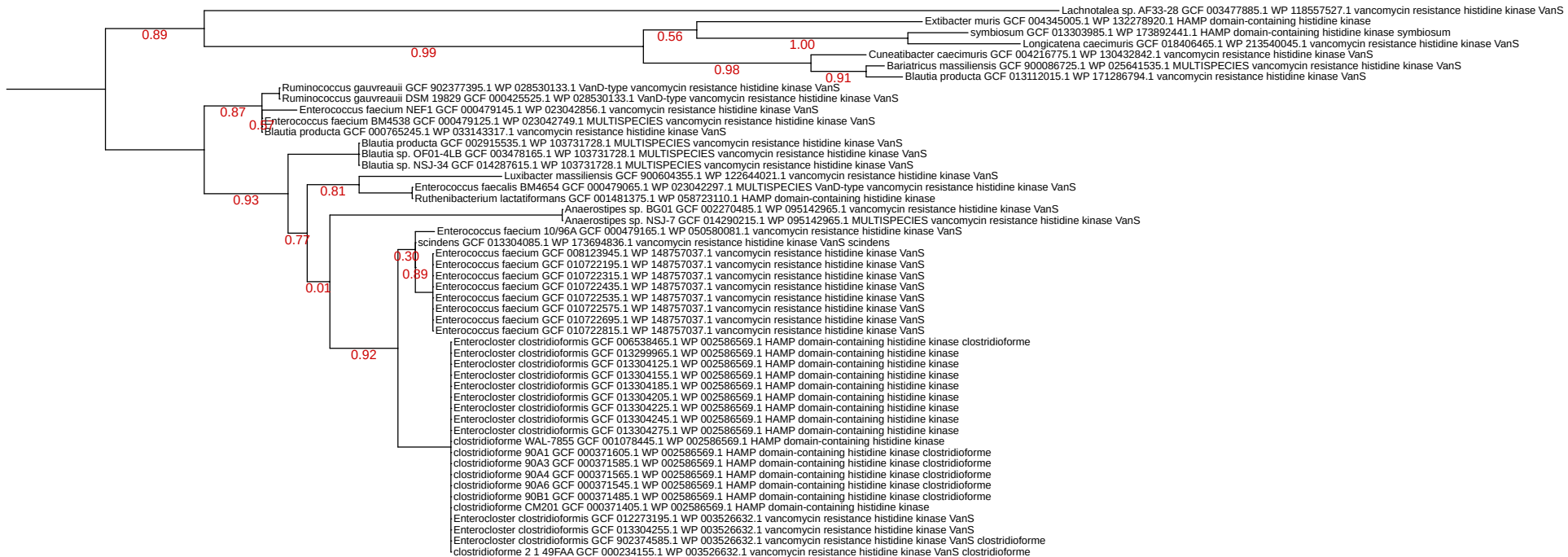

Supplement: Supplementary file 1 [file antibiotics-13-00573-s001.zip › Figure S14_Extended tree of vanS-D.pdf]
