# Supplementary material for: Phylogenetic Analysis of the Genes in D-Ala-D-Lactate Synthesizing Glycopeptide Resistance Operons: The Different Origins of Functional and Regulatory Genes
Source: Antibiotics (Basel). 2024 Jun 21;13(7):573. doi: 10.3390/antibiotics13070573 (PMC11273654; doi:10.3390/antibiotics13070573)

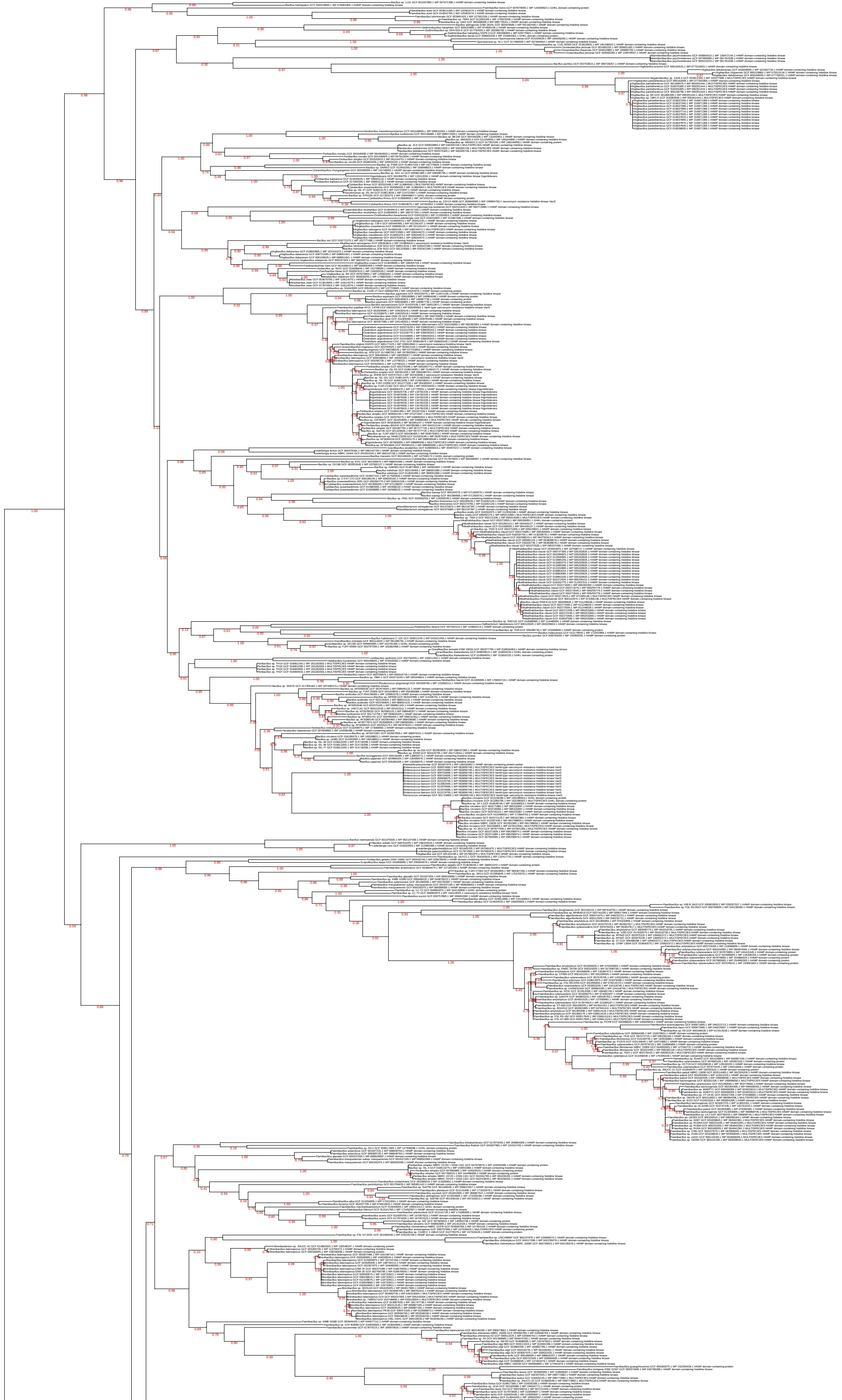

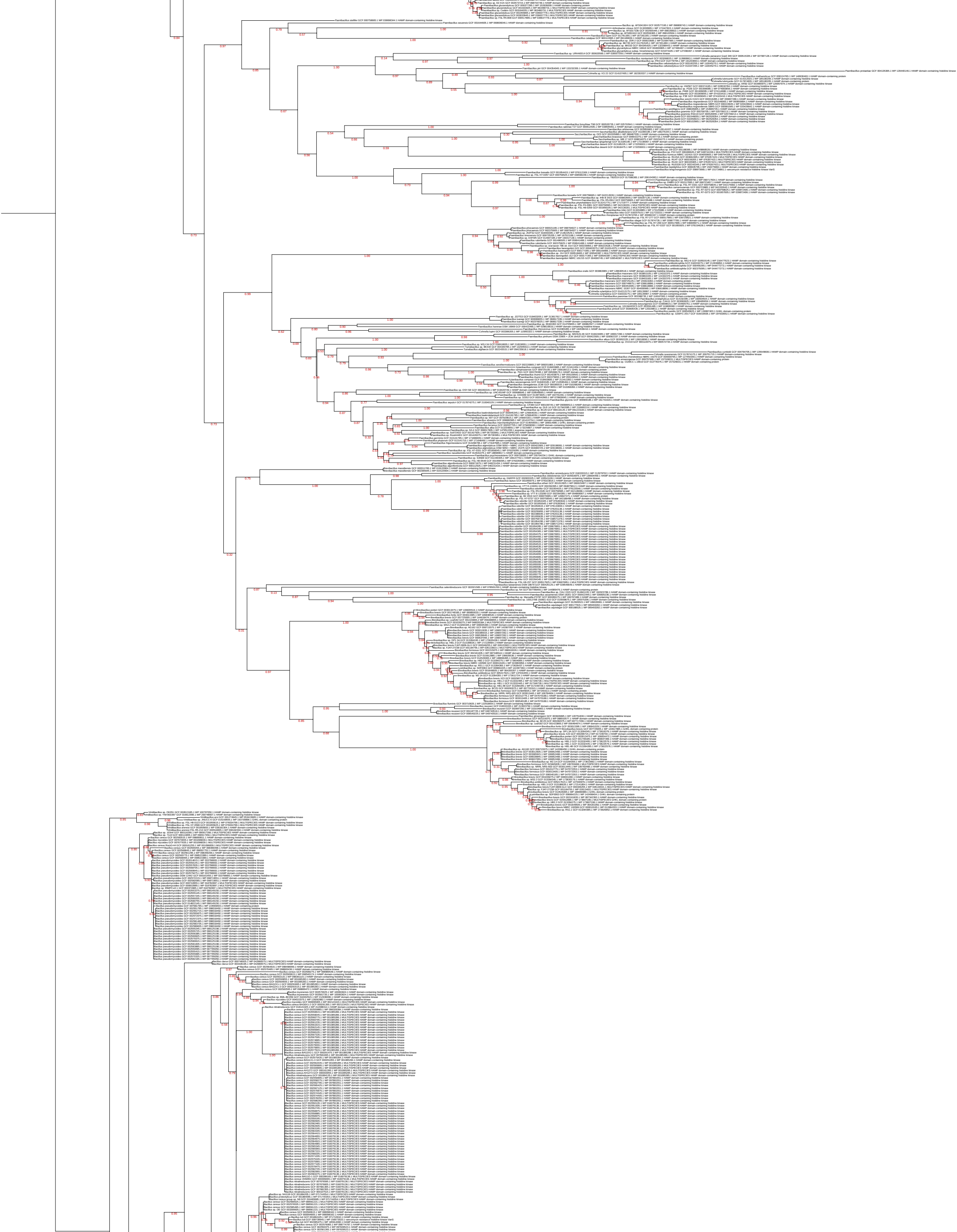

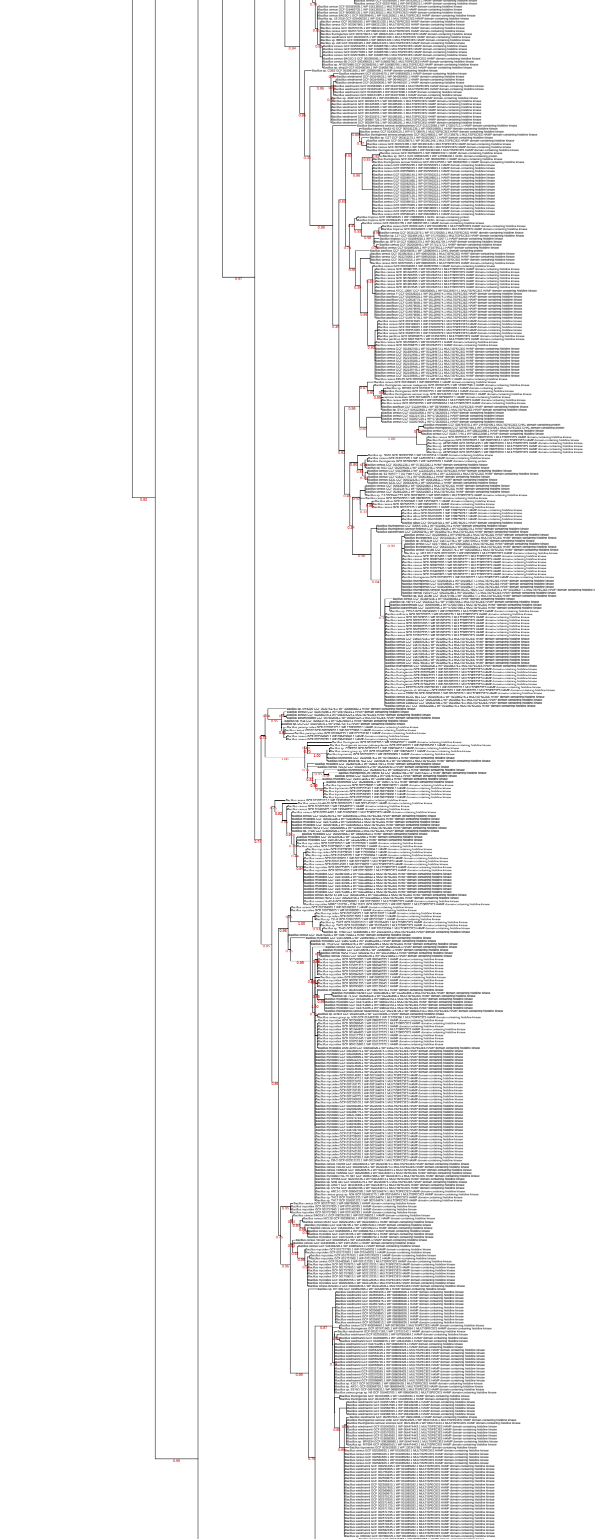

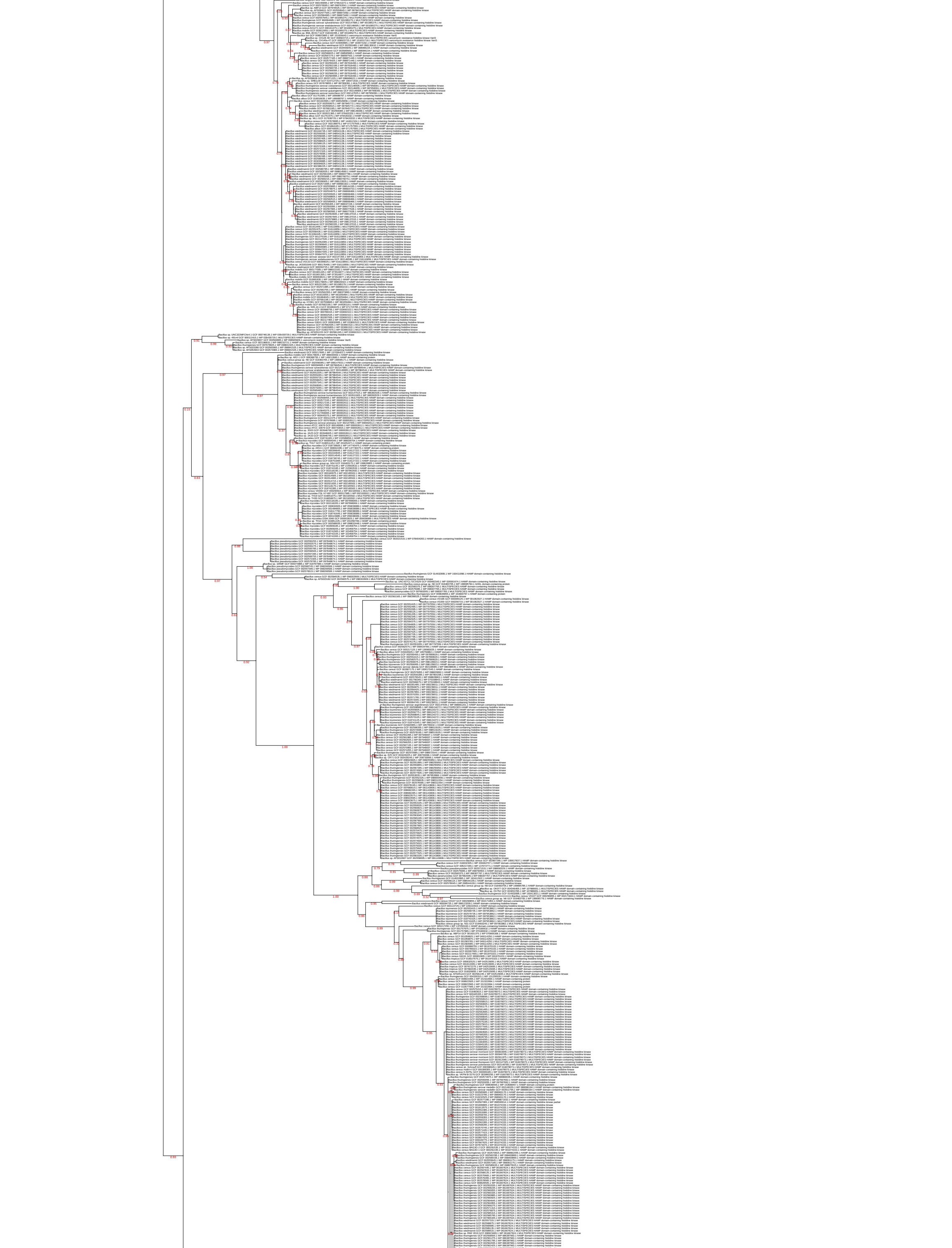

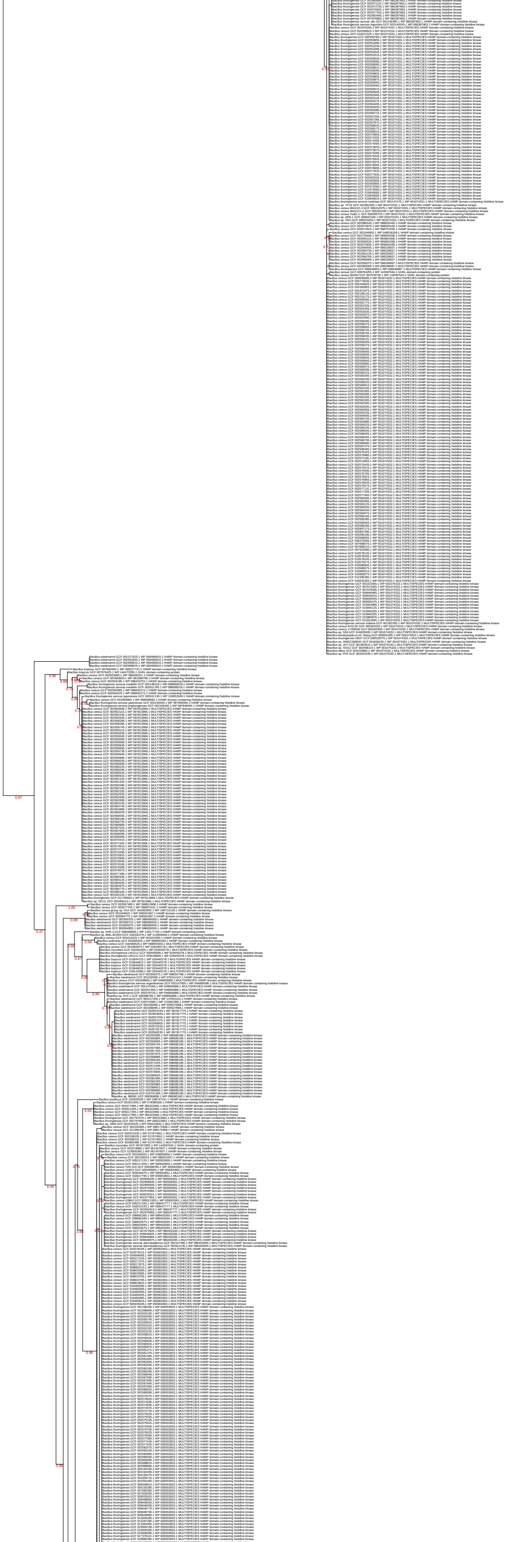

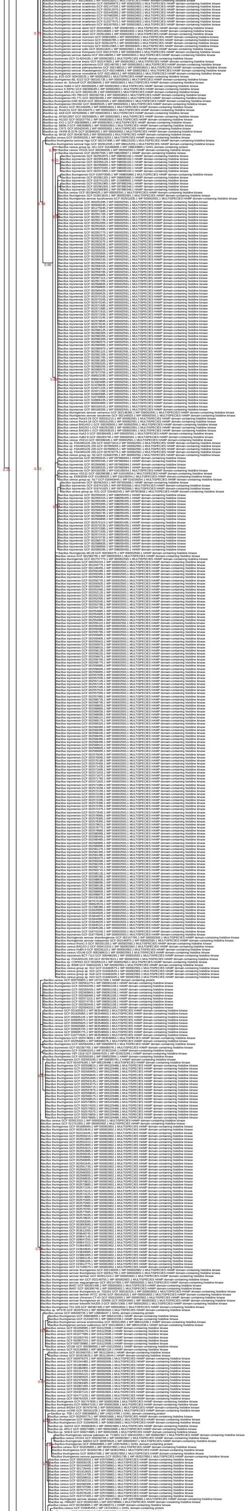

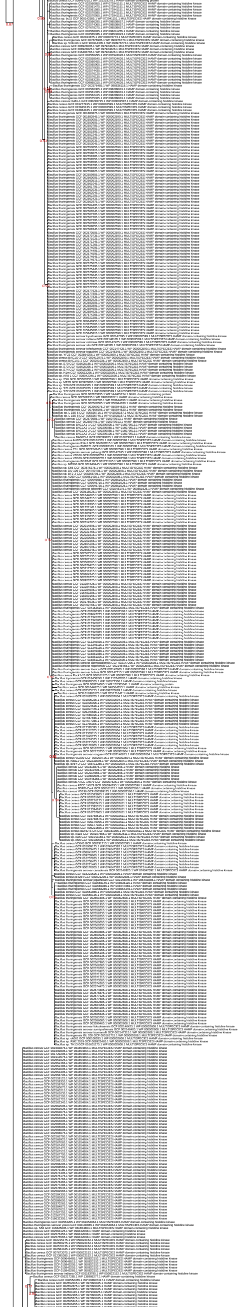

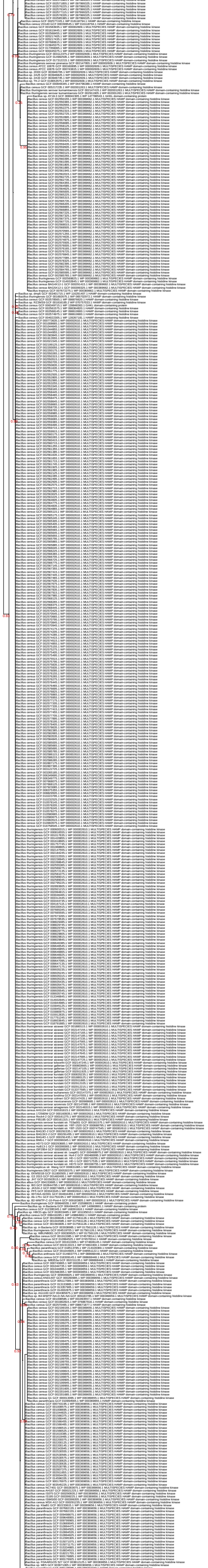

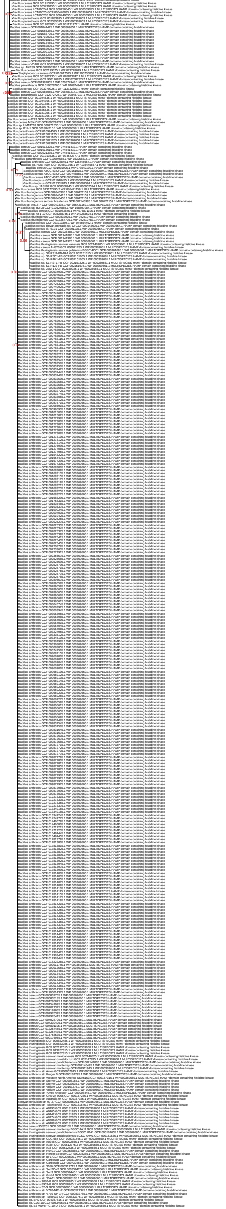

Supplement: Supplementary file 1 [file FigureS12_Extended tree of vanS-M.pdf › FigureS12_Extended tree of vanS-M.pdf]
