# Supplementary material for: Phylogenetic Analysis of the Genes in D-Ala-D-Lactate Synthesizing Glycopeptide Resistance Operons: The Different Origins of Functional and Regulatory Genes
Source: Antibiotics (Basel). 2024 Jun 21;13(7):573. doi: 10.3390/antibiotics13070573 (PMC11273654; doi:10.3390/antibiotics13070573)
Supplement: Supplementary file 1 [file FigureS1_Extended tree of vanAB.pdf › FigureS1_Extended tree of vanAB.pdf]

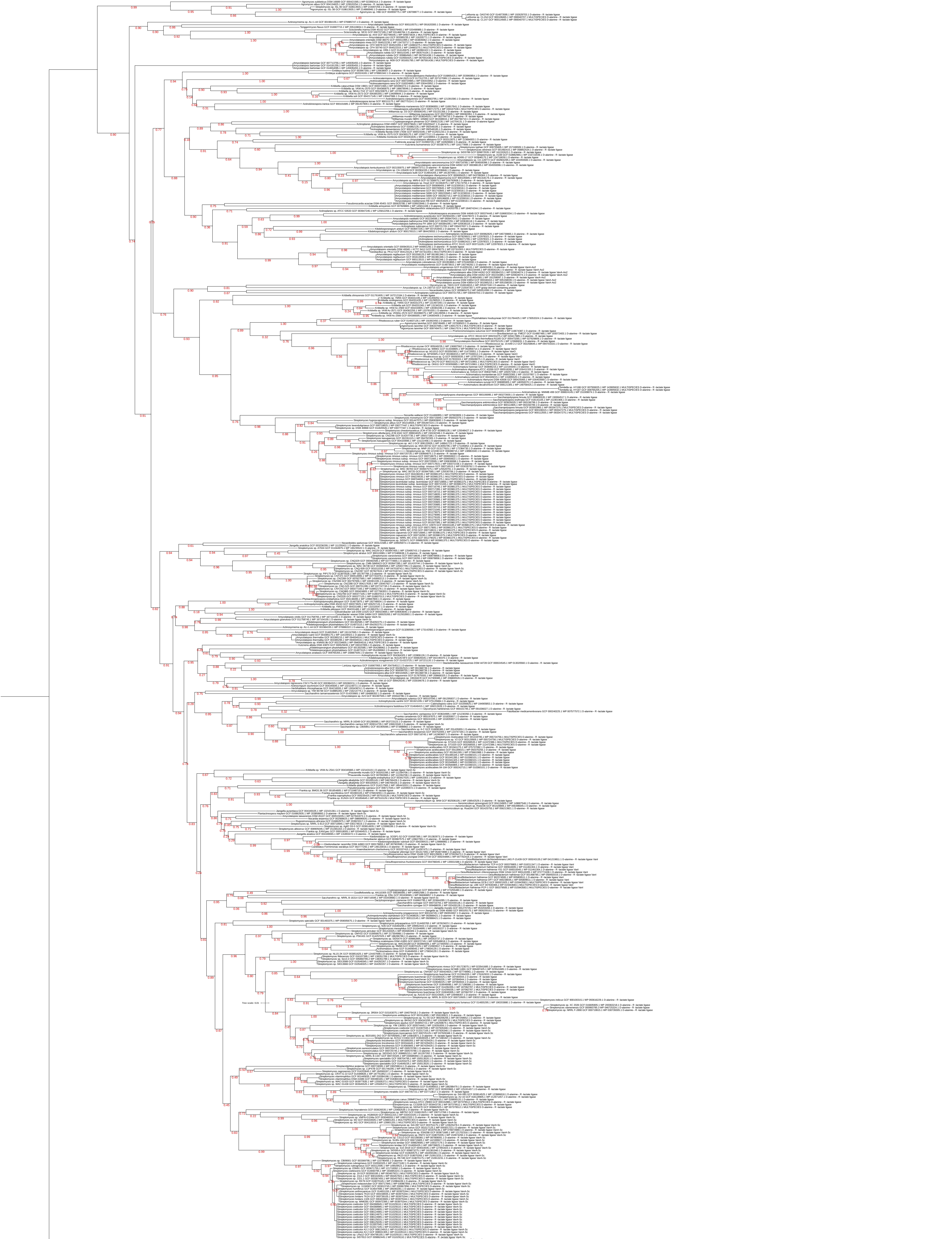
Tree scale: 0.01

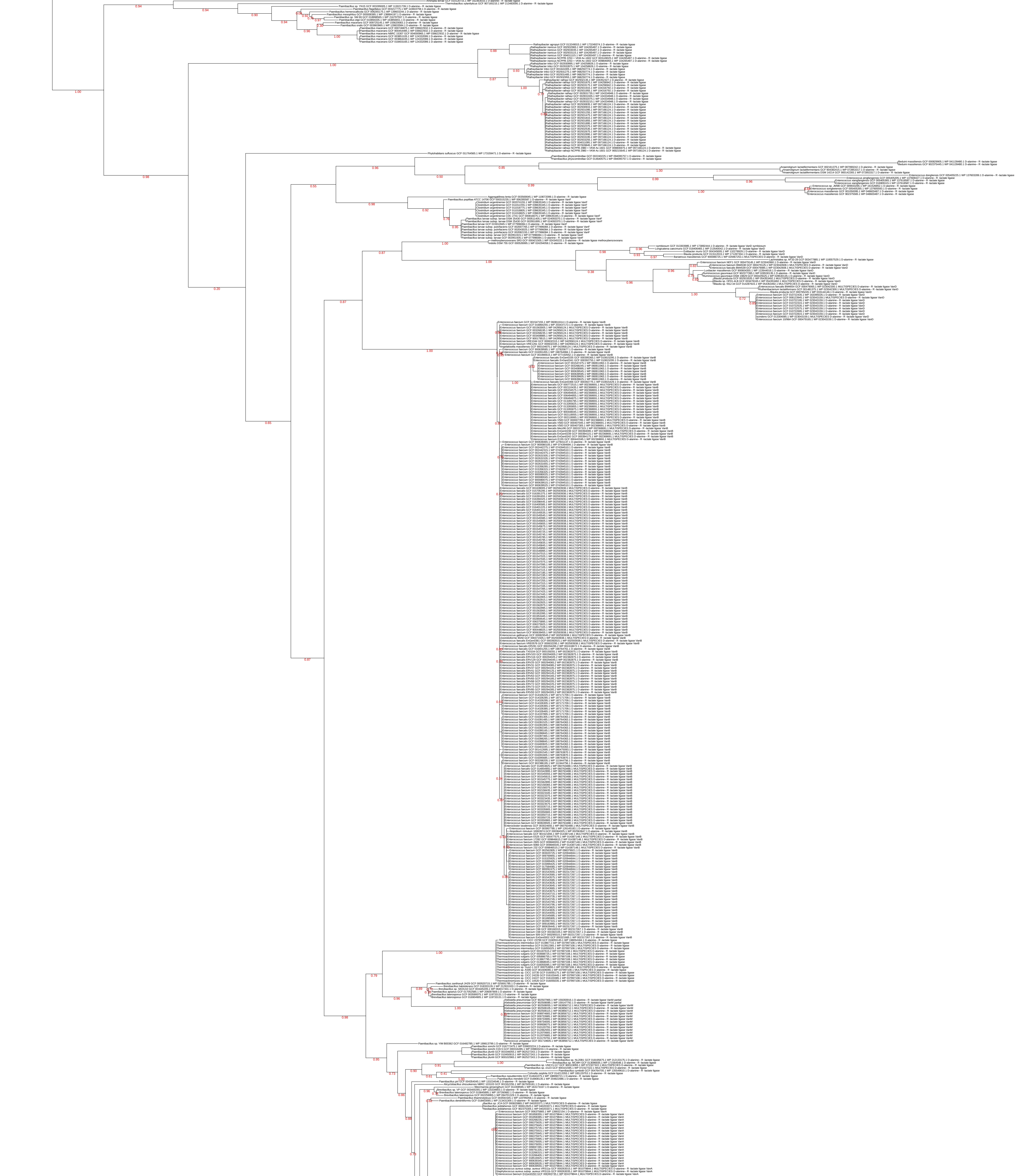

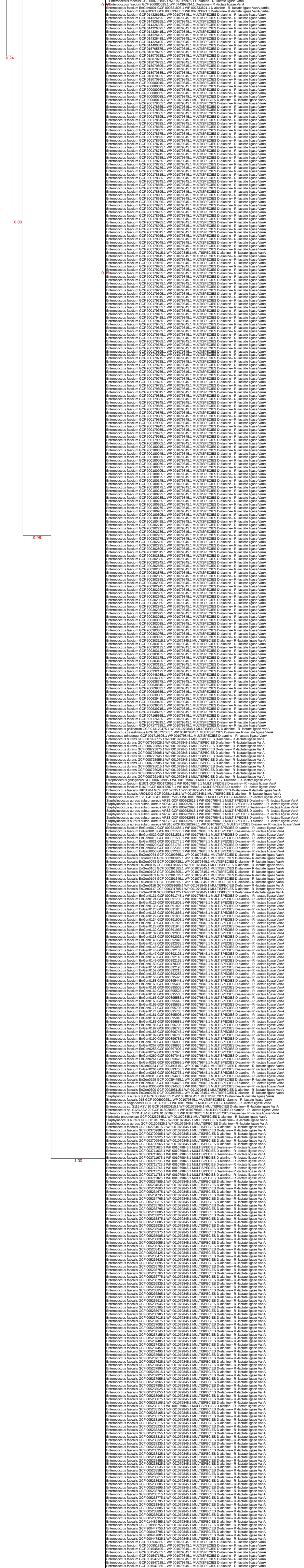
0.80
0.98
1.00

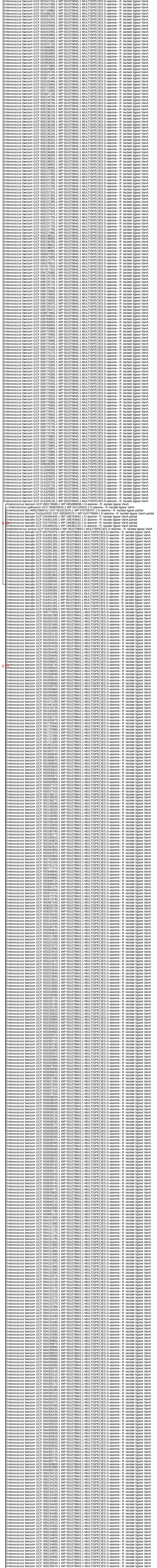

Enterococcus faecium GCF 005235115.1 WP 001079843.1 MULTISPECIES D-alanine-- R -lactate ligase VanA
Enterococcus faecium GCF 005235275.1 WP 001079843.1 MULTISPECIES D-alanine-- R -lactate ligase VanA
Enterococcus faecium GCF 005235285.1 WP 001079843.1 MULTISPECIES D-alanine-- R -lactate ligase VanA
Enterococcus faecium GCF 005235295.1 WP 001079843.1 MULTISPECIES D-alanine-- R -lactate ligase VanA
Enterococcus faecium GCF 005235305.1 WP 001079843.1 MULTISPECIES D-alanine-- R -lactate ligase VanA
Enterococcus faecium GCF 005235345.1 WP 001079843.1 MULTISPECIES D-alanine-- R -lactate ligase VanA
Enterococcus faecium GCF 005235375.1 WP 001079843.1 MULTISPECIES D-alanine-- R -lactate ligase VanA
Enterococcus faecium GCF 005235385.1 WP 001079843.1 MULTISPECIES D-alanine-- R -lactate ligase VanA
Enterococcus faecium GCF 005235395.1 WP 001079843.1 MULTISPECIES D-alanine-- R -lactate ligase VanA
Enterococcus faecium GCF 005235445.1 WP 001079843.1 MULTISPECIES D-alanine-- R -lactate ligase VanA
Enterococcus faecium GCF 005235465.1 WP 001079843.1 MULTISPECIES D-alanine-- R -lactate ligase VanA
Enterococcus faecium GCF 005235475.1 WP 001079843.1 MULTISPECIES D-alanine-- R -lactate ligase VanA
Enterococcus faecium GCF 005235495.1 WP 001079843.1 MULTISPECIES D-alanine-- R -lactate ligase VanA
Enterococcus faecium GCF 005235535.1 WP 001079843.1 MULTISPECIES D-alanine-- R -lactate ligase VanA
Enterococcus faecium GCF 005235565.1 WP 001079843.1 MULTISPECIES D-alanine-- R -lactate ligase VanA
Enterococcus faecium GCF 005235575.1 WP 001079843.1 MULTISPECIES D-alanine-- R -lactate ligase VanA
Enterococcus faecium GCF 005235585.1 WP 001079843.1 MULTISPECIES D-alanine-- R -lactate ligase VanA
Enterococcus faecium GCF 005235635.1 WP 001079843.1 MULTISPECIES D-alanine-- R -lactate ligase VanA
Enterococcus faecium GCF 005235645.1 WP 001079843.1 MULTISPECIES D-alanine-- R -lactate ligase VanA
Enterococcus faecium GCF 005235655.1 WP 001079843.1 MULTISPECIES D-alanine-- R -lactate ligase VanA
Enterococcus faecium GCF 005235665.1 WP 001079843.1 MULTISPECIES D-alanine-- R -lactate ligase VanA
Enterococcus faecium GCF 005235685.1 WP 001079843.1 MULTISPECIES D-alanine-- R -lactate ligase VanA
Enterococcus faecium GCF 005235725.1 WP 001079843.1 MULTISPECIES D-alanine-- R -lactate ligase VanA
Enterococcus faecium GCF 005235735.1 WP 001079843.1 MULTISPECIES D-alanine-- R -lactate ligase VanA
Enterococcus faecium GCF 005235965.1 WP 001079843.1 MULTISPECIES D-alanine-- R -lactate ligase VanA
Enterococcus faecium GCF 005287905.1 WP 001079843.1 MULTISPECIES D-alanine-- R -lactate ligase VanA
Enterococcus faecium GCF 005886545.1 WP 001079843.1 MULTISPECIES D-alanine-- R -lactate ligase VanA
Enterococcus faecium GCF 005886655.1 WP 001079843.1 MULTISPECIES D-alanine-- R -lactate ligase VanA
Enterococcus faecium GCF 005886715.1 WP 001079843.1 MULTISPECIES D-alanine-- R -lactate ligase VanA
Enterococcus faecium GCF 005886735.1 WP 001079843.1 MULTISPECIES D-alanine-- R -lactate ligase VanA
Enterococcus faecium GCF 005952885.1 WP 001079843.1 MULTISPECIES D-alanine-- R -lactate ligase VanA
Enterococcus faecium GCF 006375615.1 WP 001079843.1 MULTISPECIES D-alanine-- R -lactate ligase VanA
Enterococcus faecium GCF 006375855.1 WP 001079843.1 MULTISPECIES D-alanine-- R -lactate ligase VanA
Enterococcus faecium GCF 008131145.1 WP 001079843.1 MULTISPECIES D-alanine-- R -lactate ligase VanA
Enterococcus faecium GCF 008131155.1 WP 001079843.1 MULTISPECIES D-alanine-- R -lactate ligase VanA
Enterococcus faecium GCF 008131195.1 WP 001079843.1 MULTISPECIES D-alanine-- R -lactate ligase VanA
Enterococcus faecium GCF 008131205.1 WP 001079843.1 MULTISPECIES D-alanine-- R -lactate ligase VanA
Enterococcus faecium GCF 008131275.1 WP 001079843.1 MULTISPECIES D-alanine-- R -lactate ligase VanA
Enterococcus faecium GCF 008131285.1 WP 001079843.1 MULTISPECIES D-alanine-- R -lactate ligase VanA
Enterococcus faecium GCF 008131295.1 WP 001079843.1 MULTISPECIES D-alanine-- R -lactate ligase VanA
Enterococcus faecium GCF 008131305.1 WP 001079843.1 MULTISPECIES D-alanine-- R -lactate ligase VanA
Enterococcus faecium GCF 008131355.1 WP 001079843.1 MULTISPECIES D-alanine-- R -lactate ligase VanA
Enterococcus faecium GCF 008131365.1 WP 001079843.1 MULTISPECIES D-alanine-- R -lactate ligase VanA
Enterococcus faecium GCF 008131375.1 WP 001079843.1 MULTISPECIES D-alanine-- R -lactate ligase VanA
Enterococcus faecium GCF 008131385.1 WP 001079843.1 MULTISPECIES D-alanine-- R -lactate ligase VanA
Enterococcus faecium GCF 008131435.1 WP 001079843.1 MULTISPECIES D-alanine-- R -lactate ligase VanA
Enterococcus faecium GCF 008131445.1 WP 001079843.1 MULTISPECIES D-alanine-- R -lactate ligase VanA
Enterococcus faecium GCF 008131455.1 WP 001079843.1 MULTISPECIES D-alanine-- R -lactate ligase VanA
Enterococcus faecium GCF 008131485.1 WP 001079843.1 MULTISPECIES D-alanine-- R -lactate ligase VanA
Enterococcus faecium GCF 008131515.1 WP 001079843.1 MULTISPECIES D-alanine-- R -lactate ligase VanA
Enterococcus faecium GCF 008131525.1 WP 001079843.1 MULTISPECIES D-alanine-- R -lactate ligase VanA
Enterococcus faecium GCF 008131535.1 WP 001079843.1 MULTISPECIES D-alanine-- R -lactate ligase VanA
Enterococcus faecium GCF 008131565.1 WP 001079843.1 MULTISPECIES D-alanine-- R -lactate ligase VanA
Enterococcus faecium GCF 008131595.1 WP 001079843.1 MULTISPECIES D-alanine-- R -lactate ligase VanA
Enterococcus faecium GCF 008131615.1 WP 001079843.1 MULTISPECIES D-alanine-- R -lactate ligase VanA
Enterococcus faecium GCF 008131625.1 WP 001079843.1 MULTISPECIES D-alanine-- R -lactate ligase VanA
Enterococcus faecium GCF 008131645.1 WP 001079843.1 MULTISPECIES D-alanine-- R -lactate ligase VanA
Enterococcus faecium GCF 008131675.1 WP 001079843.1 MULTISPECIES D-alanine-- R -lactate ligase VanA
Enterococcus faecium GCF 008131685.1 WP 001079843.1 MULTISPECIES D-alanine-- R -lactate ligase VanA
Enterococcus faecium GCF 008131705.1 WP 001079843.1 MULTISPECIES D-alanine-- R -lactate ligase VanA
Enterococcus faecium GCF 008131725.1 WP 001079843.1 MULTISPECIES D-alanine-- R -lactate ligase VanA
Enterococcus faecium GCF 008131755.1 WP 001079843.1 MULTISPECIES D-alanine-- R -lactate ligase VanA
Enterococcus faecium GCF 008728455.1 WP 001079843.1 MULTISPECIES D-alanine-- R -lactate ligase VanA
Enterococcus faecium GCF 008921805.1 WP 001079843.1 MULTISPECIES D-alanine-- R -lactate ligase VanA
Enterococcus faecium GCF 008922005.1 WP 001079843.1 MULTISPECIES D-alanine-- R -lactate ligase VanA
Enterococcus faecium GCF 008922055.1 WP 001079843.1 MULTISPECIES D-alanine-- R -lactate ligase VanA
Enterococcus faecium GCF 009078405.1 WP 001079843.1 MULTISPECIES D-alanine-- R -lactate ligase VanA
Enterococcus faecium GCF 009078415.1 WP 001079843.1 MULTISPECIES D-alanine-- R -lactate ligase VanA
Enterococcus faecium GCF 009183865.1 WP 001079843.1 MULTISPECIES D-alanine-- R -lactate ligase VanA
Enterococcus faecium GCF 009183885.1 WP 001079843.1 MULTISPECIES D-alanine-- R -lactate ligase VanA
Enterococcus faecium GCF 009183895.1 WP 001079843.1 MULTISPECIES D-alanine-- R -lactate ligase VanA
Enterococcus faecium GCF 009183945.1 WP 001079843.1 MULTISPECIES D-alanine-- R -lactate ligase VanA
Enterococcus faecium GCF 009183955.1 WP 001079843.1 MULTISPECIES D-alanine-- R -lactate ligase VanA
Enterococcus faecium GCF 009184005.1 WP 001079843.1 MULTISPECIES D-alanine-- R -lactate ligase VanA
Enterococcus faecium GCF 009184015.1 WP 001079843.1 MULTISPECIES D-alanine-- R -lactate ligase VanA
Enterococcus faecium GCF 009184035.1 WP 001079843.1 MULTISPECIES D-alanine-- R -lactate ligase VanA
Enterococcus faecium GCF 009184055.1 WP 001079843.1 MULTISPECIES D-alanine-- R -lactate ligase VanA
Enterococcus faecium GCF 009184075.1 WP 001079843.1 MULTISPECIES D-alanine-- R -lactate ligase VanA
Enterococcus faecium GCF 009184105.1 WP 001079843.1 MULTISPECIES D-alanine-- R -lactate ligase VanA
Enterococcus faecium GCF 009184115.1 WP 001079843.1 MULTISPECIES D-alanine-- R -lactate ligase VanA
Enterococcus faecium GCF 009184145.1 WP 001079843.1 MULTISPECIES D-alanine-- R -lactate ligase VanA
Enterococcus faecium GCF 009184155.1 WP 001079843.1 MULTISPECIES D-alanine-- R -lactate ligase VanA
Enterococcus faecium GCF 009184165.1 WP 001079843.1 MULTISPECIES D-alanine-- R -lactate ligase VanA
Enterococcus faecium GCF 009184205.1 WP 001079843.1 MULTISPECIES D-alanine-- R -lactate ligase VanA
Enterococcus faecium GCF 009184225.1 WP 001079843.1 MULTISPECIES D-alanine-- R -lactate ligase VanA
Enterococcus faecium GCF 009184235.1 WP 001079843.1 MULTISPECIES D-alanine-- R -lactate ligase VanA
Enterococcus faecium GCF 009184255.1 WP 001079843.1 MULTISPECIES D-alanine-- R -lactate ligase VanA
Enterococcus faecium GCF 009184265.1 WP 001079843.1 MULTISPECIES D-alanine-- R -lactate ligase VanA
Enterococcus faecium GCF 009184285.1 WP 001079843.1 MULTISPECIES D-alanine-- R -lactate ligase VanA
Enterococcus faecium GCF 009184325.1 WP 001079843.1 MULTISPECIES D-alanine-- R -lactate ligase VanA
Enterococcus faecium GCF 009184345.1 WP 001079843.1 MULTISPECIES D-alanine-- R -lactate ligase VanA
Enterococcus faecium GCF 009734515.1 WP 001079843.1 MULTISPECIES D-alanine-- R -lactate ligase VanA
Enterococcus faecium GCF 009734805.1 WP 001079843.1 MULTISPECIES D-alanine-- R -lactate ligase VanA
Enterococcus faecium GCF 009734855.1 WP 001079843.1 MULTISPECIES D-alanine-- R -lactate ligase VanA
Enterococcus faecium GCF 009734915.1 WP 001079843.1 MULTISPECIES D-alanine-- R -lactate ligase VanA
Enterococcus faecium GCF 009734925.1 WP 001079843.1 MULTISPECIES D-alanine-- R -lactate ligase VanA
Enterococcus faecium GCF 009734975.1 WP 001079843.1 MULTISPECIES D-alanine-- R -lactate ligase VanA
Enterococcus faecium GCF 009735005.1 WP 001079843.1 MULTISPECIES D-alanine-- R -lactate ligase VanA
Enterococcus faecium GCF 009735025.1 WP 001079843.1 MULTISPECIES D-alanine-- R -lactate ligase VanA
Enterococcus faecium GCF 010119375.1 WP 001079843.1 MULTISPECIES D-alanine-- R -lactate ligase VanA
Enterococcus faecium GCF 011316145.1 WP 001079843.1 MULTISPECIES D-alanine-- R -lactate ligase VanA
Enterococcus faecium GCF 011316195.1 WP 001079843.1 MULTISPECIES D-alanine-- R -lactate ligase VanA
Enterococcus faecium GCF 011316265.1 WP 001079843.1 MULTISPECIES D-alanine-- R -lactate ligase VanA
Enterococcus faecium GCF 011386845.1 WP 001079843.1 MULTISPECIES D-alanine-- R -lactate ligase VanA
Enterococcus faecium GCF 011386855.1 WP 001079843.1 MULTISPECIES D-alanine-- R -lactate ligase VanA
Enterococcus faecium GCF 011386865.1 WP 001079843.1 MULTISPECIES D-alanine-- R -lactate ligase VanA
Enterococcus faecium GCF 012932975.2 WP 001079843.1 MULTISPECIES D-alanine-- R -lactate ligase VanA
Enterococcus faecium GCF 012932985.2 WP 001079843.1 MULTISPECIES D-alanine-- R -lactate ligase VanA
Enterococcus faecium GCF 012933055.2 WP 001079843.1 MULTISPECIES D-alanine-- R -lactate ligase VanA
Enterococcus faecium GCF 012933075.2 WP 001079843.1 MULTISPECIES D-alanine-- R -lactate ligase VanA
Enterococcus faecium GCF 012933145.1 WP 001079843.1 MULTISPECIES D-alanine-- R -lactate ligase VanA
Enterococcus faecium GCF 012933165.2 WP 001079843.1 MULTISPECIES D-alanine-- R -lactate ligase VanA
Enterococcus faecium GCF 012933185.1 WP 001079843.1 MULTISPECIES D-alanine-- R -lactate ligase VanA
Enterococcus faecium GCF 012933195.2 WP 001079843.1 MULTISPECIES D-alanine-- R -lactate ligase VanA
Enterococcus faecium GCF 012933245.2 WP 001079843.1 MULTISPECIES D-alanine-- R -lactate ligase VanA
Enterococcus faecium GCF 012933265.2 WP 001079843.1 MULTISPECIES D-alanine-- R -lactate ligase VanA
Enterococcus faecium GCF 012933275.1 WP 001079843.1 MULTISPECIES D-alanine-- R -lactate ligase VanA
Enterococcus faecium GCF 012933285.2 WP 001079843.1 MULTISPECIES D-alanine-- R -lactate ligase VanA
Enterococcus faecium GCF 012933295.2 WP 001079843.1 MULTISPECIES D-alanine-- R -lactate ligase VanA
Enterococcus faecium GCF 012952285.1 WP 001079843.1 MULTISPECIES D-alanine-- R -lactate ligase VanA
Enterococcus faecium GCF 013266375.1 WP 001079843.1 MULTISPECIES D-alanine-- R -lactate ligase VanA
Enterococcus faecium GCF 014325565.1 WP 001079843.1 MULTISPECIES D-alanine-- R -lactate ligase VanA
Enterococcus faecium GCF 014325595.1 WP 001079843.1 MULTISPECIES D-alanine-- R -lactate ligase VanA
Enterococcus faecium GCF 014325645.1 WP 001079843.1 MULTISPECIES D-alanine-- R -lactate ligase VanA
Enterococcus faecium GCF 014325665.1 WP 001079843.1 MULTISPECIES D-alanine-- R -lactate ligase VanA
Enterococcus faecium GCF 014325735.1 WP 001079843.1 MULTISPECIES D-alanine-- R -lactate ligase VanA
Enterococcus faecium GCF 014325755.1 WP 001079843.1 MULTISPECIES D-alanine-- R -lactate ligase VanA
Enterococcus faecium GCF 014325785.1 WP 001079843.1 MULTISPECIES D-alanine-- R -lactate ligase VanA
Enterococcus faecium GCF 014325825.1 WP 001079843.1 MULTISPECIES D-alanine-- R -lactate ligase VanA
Enterococcus faecium GCF 014325835.1 WP 001079843.1 MULTISPECIES D-alanine-- R -lactate ligase VanA
Enterococcus faecium GCF 014325845.1 WP 001079843.1 MULTISPECIES D-alanine-- R -lactate ligase VanA
Enterococcus faecium GCF 014325885.1 WP 001079843.1 MULTISPECIES D-alanine-- R -lactate ligase VanA
Enterococcus faecium GCF 014325925.1 WP 001079843.1 MULTISPECIES D-alanine-- R -lactate ligase VanA
Enterococcus faecium GCF 014325945.1 WP 001079843.1 MULTISPECIES D-alanine-- R -lactate ligase VanA
Enterococcus faecium GCF 014326005.1 WP 001079843.1 MULTISPECIES D-alanine-- R -lactate ligase VanA
Enterococcus faecium GCF 014326025.1 WP 001079843.1 MULTISPECIES D-alanine-- R -lactate ligase VanA
Enterococcus faecium GCF 014326035.1 WP 001079843.1 MULTISPECIES D-alanine-- R -lactate ligase VanA
Enterococcus faecium GCF 014326065.1 WP 001079843.1 MULTISPECIES D-alanine-- R -lactate ligase VanA
Enterococcus faecium GCF 014326075.1 WP 001079843.1 MULTISPECIES D-alanine-- R -lactate ligase VanA
Enterococcus faecium GCF 014326085.1 WP 001079843.1 MULTISPECIES D-alanine-- R -lactate ligase VanA
Enterococcus faecium GCF 014326145.1 WP 001079843.1 MULTISPECIES D-alanine-- R -lactate ligase VanA
Enterococcus faecium GCF 014326245.1 WP 001079843.1 MULTISPECIES D-alanine-- R -lactate ligase VanA
Enterococcus faecium GCF 014326525.1 WP 001079843.1 MULTISPECIES D-alanine-- R -lactate ligase VanA
Enterococcus faecium GCF 014326575.1 WP 001079843.1 MULTISPECIES D-alanine-- R -lactate ligase VanA
Enterococcus faecium GCF 014326585.1 WP 001079843.1 MULTISPECIES D-alanine-- R -lactate ligase VanA
Enterococcus faecium GCF 014326595.1 WP 001079843.1 MULTISPECIES D-alanine-- R -lactate ligase VanA
Enterococcus faecium GCF 014337875.1 WP 001079843.1 MULTISPECIES D-alanine-- R -lactate ligase VanA
Enterococcus faecium GCF 014874615.1 WP 001079843.1 MULTISPECIES D-alanine-- R -lactate ligase VanA
Enterococcus faecium GCF 015356225.1 WP 001079843.1 MULTISPECIES D-alanine-- R -lactate ligase VanA
Enterococcus faecium GCF 015476295.1 WP 001079843.1 MULTISPECIES D-alanine-- R -lactate ligase VanA
Enterococcus faecium GCF 015536545.1 WP 001079843.1 MULTISPECIES D-alanine-- R -lactate ligase VanA
Enterococcus faecium GCF 015536555.1 WP 001079843.1 MULTISPECIES D-alanine-- R -lactate ligase VanA
Enterococcus faecium GCF 015538405.1 WP 001079843.1 MULTISPECIES D-alanine-- R -lactate ligase VanA
Enterococcus faecium GCF 015538425.1 WP 001079843.1 MULTISPECIES D-alanine-- R -lactate ligase VanA
Enterococcus faecium GCF 015538435.1 WP 001079843.1 MULTISPECIES D-alanine-- R -lactate ligase VanA
Enterococcus faecium GCF 015538455.1 WP 001079843.1 MULTISPECIES D-alanine-- R -lactate ligase VanA
Enterococcus faecium GCF 015538475.1 WP 001079843.1 MULTISPECIES D-alanine-- R -lactate ligase VanA
Enterococcus faecium GCF 015538485.1 WP 001079843.1 MULTISPECIES D-alanine-- R -lactate ligase VanA
Enterococcus faecium GCF 015538525.1 WP 001079843.1 MULTISPECIES D-alanine-- R -lactate ligase VanA
Enterococcus faecium GCF 015538535.1 WP 001079843.1 MULTISPECIES D-alanine-- R -lactate ligase VanA
Enterococcus faecium GCF 015538545.1 WP 001079843.1 MULTISPECIES D-alanine-- R -lactate ligase VanA
Enterococcus faecium GCF 015538575.1 WP 001079843.1 MULTISPECIES D-alanine-- R -lactate ligase VanA
Enterococcus faecium GCF 015538595.1 WP 001079843.1 MULTISPECIES D-alanine-- R -lactate ligase VanA
Enterococcus faecium GCF 015538625.1 WP 001079843.1 MULTISPECIES D-alanine-- R -lactate ligase VanA
Enterococcus faecium GCF 015538635.1 WP 001079843.1 MULTISPECIES D-alanine-- R -lactate ligase VanA
Enterococcus faecium GCF 015538645.1 WP 001079843.1 MULTISPECIES D-alanine-- R -lactate ligase VanA
Enterococcus faecium GCF 015538695.1 WP 001079843.1 MULTISPECIES D-alanine-- R -lactate ligase VanA
Enterococcus faecium GCF 015538745.1 WP 001079843.1 MULTISPECIES D-alanine-- R -lactate ligase VanA
Enterococcus faecium GCF 015538785.1 WP 001079843.1 MULTISPECIES D-alanine-- R -lactate ligase VanA
Enterococcus faecium GCF 015538825.1 WP 001079843.1 MULTISPECIES D-alanine-- R -lactate ligase VanA
Enterococcus faecium GCF 015538845.1 WP 001079843.1 MULTISPECIES D-alanine-- R -lactate ligase VanA
Enterococcus faecium GCF 015538855.1 WP 001079843.1 MULTISPECIES D-alanine-- R -lactate ligase VanA
Enterococcus faecium GCF 015538865.1 WP 001079843.1 MULTISPECIES D-alanine-- R -lactate ligase VanA
Enterococcus faecium GCF 015538905.1 WP 001079843.1 MULTISPECIES D-alanine-- R -lactate ligase VanA
Enterococcus faecium GCF 015538925.1 WP 001079843.1 MULTISPECIES D-alanine-- R -lactate ligase VanA
Enterococcus faecium GCF 015538935.1 WP 001079843.1 MULTISPECIES D-alanine-- R -lactate ligase VanA
Enterococcus faecium GCF 015538945.1 WP 001079843.1 MULTISPECIES D-alanine-- R -lactate ligase VanA
Enterococcus faecium GCF 015538975.1 WP 001079843.1 MULTISPECIES D-alanine-- R -lactate ligase VanA
Enterococcus faecium GCF 015538985.1 WP 001079843.1 MULTISPECIES D-alanine-- R -lactate ligase VanA
Enterococcus faecium GCF 015551885.1 WP 001079843.1 MULTISPECIES D-alanine-- R -lactate ligase VanA
Enterococcus faecium GCF 015671075.1 WP 001079843.1 MULTISPECIES D-alanine-- R -lactate ligase VanA
Enterococcus faecium GCF 015703745.1 WP 001079843.1 MULTISPECIES D-alanine-- R -lactate ligase VanA
Enterococcus faecium GCF 015705215.1 WP 001079843.1 MULTISPECIES D-alanine-- R -lactate ligase VanA
Enterococcus faecium GCF 015705235.1 WP 001079843.1 MULTISPECIES D-alanine-- R -lactate ligase VanA
Enterococcus faecium GCF 015705245.1 WP 001079843.1 MULTISPECIES D-alanine-- R -lactate ligase VanA
Enterococcus faecium GCF 015705255.1 WP 001079843.1 MULTISPECIES D-alanine-- R -lactate ligase VanA
Enterococcus faecium GCF 015705315.1 WP 001079843.1 MULTISPECIES D-alanine-- R -lactate ligase VanA
Enterococcus faecium GCF 015705325.1 WP 001079843.1 MULTISPECIES D-alanine-- R -lactate ligase VanA
Enterococcus faecium GCF 015705345.1 WP 001079843.1 MULTISPECIES D-alanine-- R -lactate ligase VanA
Enterococcus faecium GCF 015705355.1 WP 001079843.1 MULTISPECIES D-alanine-- R -lactate ligase VanA
Enterococcus faecium GCF 015705395.1 WP 001079843.1 MULTISPECIES D-alanine-- R -lactate ligase VanA
Enterococcus faecium GCF 015705415.1 WP 001079843.1 MULTISPECIES D-alanine-- R -lactate ligase VanA
Enterococcus faecium GCF 015705425.1 WP 001079843.1 MULTISPECIES D-alanine-- R -lactate ligase VanA
Enterococcus faecium GCF 015705435.1 WP 001079843.1 MULTISPECIES D-alanine-- R -lactate ligase VanA
Enterococcus faecium GCF 015705475.1 WP 001079843.1 MULTISPECIES D-alanine-- R -lactate ligase VanA
Enterococcus faecium GCF 015705515.1 WP 001079843.1 MULTISPECIES D-alanine-- R -lactate ligase VanA
Enterococcus faecium GCF 015705535.1 WP 001079843.1 MULTISPECIES D-alanine-- R -lactate ligase VanA
Enterococcus faecium GCF 015705545.1 WP 001079843.1 MULTISPECIES D-alanine-- R -lactate ligase VanA
Enterococcus faecium GCF 015705565.1 WP 001079843.1 MULTISPECIES D-alanine-- R -lactate ligase VanA
Enterococcus faecium GCF 015705595.1 WP 001079843.1 MULTISPECIES D-alanine-- R -lactate ligase VanA
Enterococcus faecium GCF 015705615.1 WP 001079843.1 MULTISPECIES D-alanine-- R -lactate ligase VanA
Enterococcus faecium GCF 015705635.1 WP 001079843.1 MULTISPECIES D-alanine-- R -lactate ligase VanA
Enterococcus faecium GCF 015705645.1 WP 001079843.1 MULTISPECIES D-alanine-- R -lactate ligase VanA
Enterococcus faecium GCF 015705655.1 WP 001079843.1 MULTISPECIES D-alanine-- R -lactate ligase VanA
Enterococcus faecium GCF 015705695.1 WP 001079843.1 MULTISPECIES D-alanine-- R -lactate ligase VanA
Enterococcus faecium GCF 015705715.1 WP 001079843.1 MULTISPECIES D-alanine-- R -lactate ligase VanA
Enterococcus faecium GCF 015705735.1 WP 001079843.1 MULTISPECIES D-alanine-- R -lactate ligase VanA
Enterococcus faecium GCF 015705745.1 WP 001079843.1 MULTISPECIES D-alanine-- R -lactate ligase VanA
Enterococcus faecium GCF 015705765.1 WP 001079843.1 MULTISPECIES D-alanine-- R -lactate ligase VanA
Enterococcus faecium GCF 015705795.1 WP 001079843.1 MULTISPECIES D-alanine-- R -lactate ligase VanA
Enterococcus faecium GCF 015705815.1 WP 001079843.1 MULTISPECIES D-alanine-- R -lactate ligase VanA
Enterococcus faecium GCF 015705825.1 WP 001079843.1 MULTISPECIES D-alanine-- R -lactate ligase VanA
Enterococcus faecium GCF 015705855.1 WP 001079843.1 MULTISPECIES D-alanine-- R -lactate ligase VanA
Enterococcus faecium GCF 015705895.1 WP 001079843.1 MULTISPECIES D-alanine-- R -lactate ligase VanA
Enterococcus faecium GCF 015705915.1 WP 001079843.1 MULTISPECIES D-alanine-- R -lactate ligase VanA
Enterococcus faecium GCF 015705935.1 WP 001079843.1 MULTISPECIES D-alanine-- R -lactate ligase VanA
Enterococcus faecium GCF 015705945.1 WP 001079843.1 MULTISPECIES D-alanine-- R -lactate ligase VanA
Enterococcus faecium GCF 015705975.1 WP 001079843.1 MULTISPECIES D-alanine-- R -lactate ligase VanA
Enterococcus faecium GCF 015705995.1 WP 001079843.1 MULTISPECIES D-alanine-- R -lactate ligase VanA
Enterococcus faecium GCF 015706005.1 WP 001079843.1 MULTISPECIES D-alanine-- R -lactate ligase VanA
Enterococcus faecium GCF 015706035.1 WP 001079843.1 MULTISPECIES D-alanine-- R -lactate ligase VanA
Enterococcus faecium GCF 015706045.1 WP 001079843.1 MULTISPECIES D-alanine-- R -lactate ligase VanA
Enterococcus faecium GCF 015706055.1 WP 001079843.1 MULTISPECIES D-alanine-- R -lactate ligase VanA
Enterococcus faecium GCF 015706095.1 WP 001079843.1 MULTISPECIES D-alanine-- R -lactate ligase VanA
Enterococcus faecium GCF 015706115.1 WP 001079843.1 MULTISPECIES D-alanine-- R -lactate ligase VanA
Enterococcus faecium GCF 015706125.1 WP 001079843.1 MULTISPECIES D-alanine-- R -lactate ligase VanA
Enterococcus faecium GCF 015706135.1 WP 001079843.1 MULTISPECIES D-alanine-- R -lactate ligase VanA
Enterococcus faecium GCF 015706145.1 WP 001079843.1 MULTISPECIES D-alanine-- R -lactate ligase VanA
Enterococcus faecium GCF 015706195.1 WP 001079843.1 MULTISPECIES D-alanine-- R -lactate ligase VanA
Enterococcus faecium GCF 015706205.1 WP 001079843.1 MULTISPECIES D-alanine-- R -lactate ligase VanA
Enterococcus faecium GCF 015706225.1 WP 001079843.1 MULTISPECIES D-alanine-- R -lactate ligase VanA
Enterococcus faecium GCF 015706235.1 WP 001079843.1 MULTISPECIES D-alanine-- R -lactate ligase VanA
Enterococcus faecium GCF 015706255.1 WP 001079843.1 MULTISPECIES D-alanine-- R -lactate ligase VanA
Enterococcus faecium GCF 015706295.1 WP 001079843.1 MULTISPECIES D-alanine-- R -lactate ligase VanA
Enterococcus faecium GCF 015706305.1 WP 001079843.1 MULTISPECIES D-alanine-- R -lactate ligase VanA
Enterococcus faecium GCF 015706335.1 WP 001079843.1 MULTISPECIES D-alanine-- R -lactate ligase VanA
Enterococcus faecium GCF 015706345.1 WP 001079843.1 MULTISPECIES D-alanine-- R -lactate ligase VanA
Enterococcus faecium GCF 015706355.1 WP 001079843.1 MULTISPECIES D-alanine-- R -lactate ligase VanA
Enterococcus faecium GCF 015706395.1 WP 001079843.1 MULTISPECIES D-alanine-- R -lactate ligase VanA
Enterococcus faecium GCF 015706415.1 WP 001079843.1 MULTISPECIES D-alanine-- R -lactate ligase VanA
Enterococcus faecium GCF 015706435.1 WP 001079843.1 MULTISPECIES D-alanine-- R -lactate ligase VanA
Enterococcus faecium GCF 015706445.1 WP 001079843.1 MULTISPECIES D-alanine-- R -lactate ligase VanA
Enterococcus faecium GCF 015706455.1 WP 001079843.1 MULTISPECIES D-alanine-- R -lactate ligase VanA
Enterococcus faecium GCF 015706495.1 WP 001079843.1 MULTISPECIES D-alanine-- R -lactate ligase VanA
Enterococcus faecium GCF 015706515.1 WP 001079843.1 MULTISPECIES D-alanine-- R -lactate ligase VanA
Enterococcus faecium GCF 015706535.1 WP 001079843.1 MULTISPECIES D-alanine-- R -lactate ligase VanA
Enterococcus faecium GCF 015706555.1 WP 001079843.1 MULTISPECIES D-alanine-- R -lactate ligase VanA
Enterococcus faecium GCF 015999605.1 WP 001079843.1 MULTISPECIES D-alanine-- R -lactate ligase VanA
Enterococcus faecium GCF 016126675.1 WP 001079843.1 MULTISPECIES D-alanine-- R -lactate ligase VanA
Enterococcus faecium GCF 016404975.1 WP 001079843.1 MULTISPECIES D-alanine-- R -lactate ligase VanA
Enterococcus faecium GCF 016405005.1 WP 001079843.1 MULTISPECIES D-alanine-- R -lactate ligase VanA
Enterococcus faecium GCF 016405025.1 WP 001079843.1 MULTISPECIES D-alanine-- R -lactate ligase VanA
Enterococcus faecium GCF 016405035.1 WP 001079843.1 MULTISPECIES D-alanine-- R -lactate ligase VanA
Enterococcus faecium GCF 016405065.1 WP 001079843.1 MULTISPECIES D-alanine-- R -lactate ligase VanA
Enterococcus faecium GCF 016405075.1 WP 001079843.1 MULTISPECIES D-alanine-- R -lactate ligase VanA
Enterococcus faecium GCF 016405085.1 WP 001079843.1 MULTISPECIES D-alanine-- R -lactate ligase VanA
Enterococcus faecium GCF 016405125.1 WP 001079843.1 MULTISPECIES D-alanine-- R -lactate ligase VanA
Enterococcus faecium GCF 016406345.1 WP 001079843.1 MULTISPECIES D-alanine-- R -lactate ligase VanA
Enterococcus faecium GCF 016406365.1 WP 001079843.1 MULTISPECIES D-alanine-- R -lactate ligase VanA
Enterococcus faecium GCF 016406385.1 WP 001079843.1 MULTISPECIES D-alanine-- R -lactate ligase VanA
Enterococcus faecium GCF 016406405.1 WP 001079843.1 MULTISPECIES D-alanine-- R -lactate ligase VanA
Enterococcus faecium GCF 016406425.1 WP 001079843.1 MULTISPECIES D-alanine-- R -lactate ligase VanA
Enterococcus faecium GCF 016406445.1 WP 001079843.1 MULTISPECIES D-alanine-- R -lactate ligase VanA
Enterococcus faecium GCF 016406465.1 WP 001079843.1 MULTISPECIES D-alanine-- R -lactate ligase VanA
Enterococcus faecium GCF 016406485.1 WP 001079843.1 MULTISPECIES D-alanine-- R -lactate ligase VanA
Enterococcus faecium GCF 016406505.1 WP 001079843.1 MULTISPECIES D-alanine-- R -lactate ligase VanA
Enterococcus faecium GCF 016406525.1 WP 001079843.1 MULTISPECIES D-alanine-- R -lactate ligase VanA
Enterococcus faecium GCF 016406545.1 WP 001079843.1 MULTISPECIES D-alanine-- R -lactate ligase VanA
Enterococcus faecium GCF 016406565.1 WP 001079843.1 MULTISPECIES D-alanine-- R -lactate ligase VanA
Enterococcus faecium GCF 016406585.1 WP 001079843.1 MULTISPECIES D-alanine-- R -lactate ligase VanA
Enterococcus faecium GCF 016406605.1 WP 001079843.1 MULTISPECIES D-alanine-- R -lactate ligase VanA
Enterococcus faecium GCF 016415025.1 WP 001079843.1 MULTISPECIES D-alanine-- R -lactate ligase VanA
Enterococcus faecium GCF 016415105.1 WP 001079843.1 MULTISPECIES D-alanine-- R -lactate ligase VanA
Enterococcus faecium GCF 016415285.1 WP 001079843.1 MULTISPECIES D-alanine-- R -lactate ligase VanA
Enterococcus faecium GCF 016415325.1 WP 001079843.1 MULTISPECIES D-alanine-- R -lactate ligase VanA
Enterococcus faecium GCF 016415345.1 WP 001079843.1 MULTISPECIES D-alanine-- R -lactate ligase VanA
Enterococcus faecium GCF 016415365.1 WP 001079843.1 MULTISPECIES D-alanine-- R -lactate ligase VanA
Enterococcus faecium GCF 016415385.1 WP 001079843.1 MULTISPECIES D-alanine-- R -lactate ligase VanA
Enterococcus faecium GCF 016415405.1 WP 001079843.1 MULTISPECIES D-alanine-- R -lactate ligase VanA
Enterococcus faecium GCF 016415425.1 WP 001079843.1 MULTISPECIES D-alanine-- R -lactate ligase VanA
Enterococcus faecium GCF 016415445.1 WP 001079843.1 MULTISPECIES D-alanine-- R -lactate ligase VanA
Enterococcus faecium GCF 016415465.1 WP 001079843.1 MULTISPECIES D-alanine-- R -lactate ligase VanA
Enterococcus faecium GCF 016415505.1 WP 001079843.1 MULTISPECIES D-alanine-- R -lactate ligase VanA
Enterococcus faecium GCF 016415545.1 WP 001079843.1 MULTISPECIES D-alanine-- R -lactate ligase VanA
Enterococcus faecium GCF 016415565.1 WP 001079843.1 MULTISPECIES D-alanine-- R -lactate ligase VanA
Enterococcus faecium GCF 016455345.1 WP 001079843.1 MULTISPECIES D-alanine-- R -lactate ligase VanA
Enterococcus faecium GCF 016484185.1 WP 001079843.1 MULTISPECIES D-alanine-- R -lactate ligase VanA
Enterococcus faecium GCF 016484285.1 WP 001079843.1 MULTISPECIES D-alanine-- R -lactate ligase VanA
Enterococcus faecium GCF 016484305.1 WP 001079843.1 MULTISPECIES D-alanine-- R -lactate ligase VanA
Enterococcus faecium GCF 016484315.1 WP 001079843.1 MULTISPECIES D-alanine-- R -lactate ligase VanA
Enterococcus faecium GCF 016484345.1 WP 001079843.1 MULTISPECIES D-alanine-- R -lactate ligase VanA
Enterococcus faecium GCF 016484425.1 WP 001079843.1 MULTISPECIES D-alanine-- R -lactate ligase VanA
Enterococcus faecium GCF 016484505.1 WP 001079843.1 MULTISPECIES D-alanine-- R -lactate ligase VanA
Enterococcus faecium GCF 017603725.1 WP 001079843.1 MULTISPECIES D-alanine-- R -lactate ligase VanA
Enterococcus faecium GCF 017815655.1 WP 001079843.1 MULTISPECIES D-alanine-- R -lactate ligase VanA
Enterococcus faecium GCF 017815675.1 WP 001079843.1 MULTISPECIES D-alanine-- R -lactate ligase VanA
Enterococcus faecium GCF 017815695.1 WP 001079843.1 MULTISPECIES D-alanine-- R -lactate ligase VanA
Enterococcus faecium GCF 017897965.1 WP 001079843.1 MULTISPECIES D-alanine-- R -lactate ligase VanA
Enterococcus faecium GCF 017898005.1 WP 001079843.1 MULTISPECIES D-alanine-- R -lactate ligase VanA
Enterococcus faecium GCF 017898025.1 WP 001079843.1 MULTISPECIES D-alanine-- R -lactate ligase VanA
Enterococcus faecium GCF 018070855.1 WP 001079843.1 MULTISPECIES D-alanine-- R -lactate ligase VanA
Enterococcus faecium GCF 018070905.1 WP 001079843.1 MULTISPECIES D-alanine-- R -lactate ligase VanA
Enterococcus faecium GCF 018219305.1 WP 001079843.1 MULTISPECIES D-alanine-- R -lactate ligase VanA
Enterococcus faecium GCF 018219325.1 WP 001079843.1 MULTISPECIES D-alanine-- R -lactate ligase VanA
Enterococcus faecium GCF 018219345.1 WP 001079843.1 MULTISPECIES D-alanine-- R -lactate ligase VanA
Enterococcus faecium GCF 018296025.1 WP 001079843.1 MULTISPECIES D-alanine-- R -lactate ligase VanA
Enterococcus faecium GCF 018531525.1 WP 001079843.1 MULTISPECIES D-alanine-- R -lactate ligase VanA
Enterococcus faecium GCF 018531545.1 WP 001079843.1 MULTISPECIES D-alanine-- R -lactate ligase VanA
Enterococcus faecium GCF 018531555.1 WP 001079843.1 MULTISPECIES D-alanine-- R -lactate ligase VanA
Enterococcus faecium GCF 018531585.1 WP 001079843.1 MULTISPECIES D-alanine-- R -lactate ligase VanA
Enterococcus faecium GCF 018531605.1 WP 001079843.1 MULTISPECIES D-alanine-- R -lactate ligase VanA
Enterococcus faecium GCF 018531625.1 WP 001079843.1 MULTISPECIES D-alanine-- R -lactate ligase VanA
Enterococcus faecium GCF 018531635.1 WP 001079843.1 MULTISPECIES D-alanine-- R -lactate ligase VanA
Enterococcus faecium GCF 018531665.1 WP 001079843.1 MULTISPECIES D-alanine-- R -lactate ligase VanA
Enterococcus faecium GCF 018531675.1 WP 001079843.1 MULTISPECIES D-alanine-- R -lactate ligase VanA
Enterococcus faecium GCF 018531705.1 WP 001079843.1 MULTISPECIES D-alanine-- R -lactate ligase VanA
Enterococcus faecium GCF 900178635.1 WP 001079843.1 MULTISPECIES D-alanine-- R -lactate ligase VanA
Enterococcus faecium GCF 900178645.1 WP 001079843.1 MULTISPECIES D-alanine-- R -lactate ligase VanA
Enterococcus faecium GCF 900179065.1 WP 001079843.1 MULTISPECIES D-alanine-- R -lactate ligase VanA
Enterococcus faecium GCF 900179075.1 WP 001079843.1 MULTISPECIES D-alanine-- R -lactate ligase VanA
Enterococcus faecium GCF 900179095.1 WP 001079843.1 MULTISPECIES D-alanine-- R -lactate ligase VanA
Enterococcus faecium GCF 900179105.1 WP 001079843.1 MULTISPECIES D-alanine-- R -lactate ligase VanA
Enterococcus faecium GCF 900179215.1 WP 001079843.1 MULTISPECIES D-alanine-- R -lactate ligase VanA
Enterococcus faecium GCF 900179385.1 WP 001079843.1 MULTISPECIES D-alanine-- R -lactate ligase VanA
Enterococcus faecium GCF 900179445.1 WP 001079843.1 MULTISPECIES D-alanine-- R -lactate ligase VanA
Enterococcus faecium GCF 900179475.1 WP 001079843.1 MULTISPECIES D-alanine-- R -lactate ligase VanA
Enterococcus faecium GCF 900179925.1 WP 001079843.1 MULTISPECIES D-alanine-- R -lactate ligase VanA
Enterococcus faecium GCF 900179935.1 WP 001079843.1 MULTISPECIES D-alanine-- R -lactate ligase VanA
Enterococcus faecium GCF 900179975.1 WP 001079843.1 MULTISPECIES D-alanine-- R -lactate ligase VanA
Enterococcus faecium GCF 900180035.1 WP 001079843.1 MULTISPECIES D-alanine-- R -lactate ligase VanA
Enterococcus faecium GCF 900180115.1 WP 001079843.1 MULTISPECIES D-alanine-- R -lactate ligase VanA
Enterococcus faecium GCF 900180165.1 WP 001079843.1 MULTISPECIES D-alanine-- R -lactate ligase VanA
Enterococcus faecium GCF 900180185.1 WP 001079843.1 MULTISPECIES D-alanine-- R -lactate ligase VanA
Enterococcus faecium GCF 900180195.1 WP 001079843.1 MULTISPECIES D-alanine-- R -lactate ligase VanA
Enterococcus faecium GCF 900180205.1 WP 001079843.1 MULTISPECIES D-alanine-- R -lactate ligase VanA
Enterococcus faecium GCF 900180245.1 WP 001079843.1 MULTISPECIES D-alanine-- R -lactate ligase VanA
Enterococcus faecium GCF 900180325.1 WP 001079843.1 MULTISPECIES D-alanine-- R -lactate ligase VanA
Enterococcus faecium GCF 900180465.1 WP 001079843.1 MULTISPECIES D-alanine-- R -lactate ligase VanA
Enterococcus faecium GCF 900303005.1 WP 001079843.1 MULTISPECIES D-alanine-- R -lactate ligase VanA
Enterococcus faecium GCF 900303045.1 WP 001079843.1 MULTISPECIES D-alanine-- R -lactate ligase VanA
Enterococcus faecium GCF 900303105.1 WP 001079843.1 MULTISPECIES D-alanine-- R -lactate ligase VanA
Enterococcus faecium GCF 900639395.1 WP 001079843.1 MULTISPECIES D-alanine-- R -lactate ligase VanA
Enterococcus faecium GCF 900639475.1 WP 001079843.1 MULTISPECIES D-alanine-- R -lactate ligase VanA
Enterococcus faecium GCF 900639485.1 WP 001079843.1 MULTISPECIES D-alanine-- R -lactate ligase VanA
Enterococcus faecium GCF 900639495.1 WP 001079843.1 MULTISPECIES D-alanine-- R -lactate ligase VanA
Enterococcus faecium GCF 900639725.1 WP 001079843.1 MULTISPECIES D-alanine-- R -lactate ligase VanA
Enterococcus avium GCF 004010255.1 WP 001079843.1 MULTISPECIES D-alanine-- R -lactate ligase VanA
Enterococcus mundtii GCF 015550885.1 WP 001079843.1 MULTISPECIES D-alanine-- R -lactate ligase VanA
Enterococcus mundtii GCF 015555705.1 WP 001079843.1 MULTISPECIES D-alanine-- R -lactate ligase VanA
Enterococcus faecium 1 230 933 GCF 000157435.1 WP 001079843.1 MULTISPECIES D-alanine-- R -lactate ligase VanA
Enterococcus faecium 1 231 502 GCF 000157535.1 WP 001079843.1 MULTISPECIES D-alanine-- R -lactate ligase VanA
Enterococcus faecalis S613 GCF 000163795.1 WP 001079843.1 MULTISPECIES D-alanine-- R -lactate ligase VanA
Enterococcus faecalis R712 GCF 000163815.1 WP 001079843.1 MULTISPECIES D-alanine-- R -lactate ligase VanA
Enterococcus faecalis R712 GCF 009662495.1 WP 001079843.1 MULTISPECIES D-alanine-- R -lactate ligase VanA
Enterococcus faecalis DAPTO 512 GCF 000148005.1 WP 001079843.1 MULTISPECIES D-alanine-- R -lactate ligase VanA
Enterococcus faecalis DAPTO 516 GCF 000147985.1 WP 001079843.1 MULTISPECIES D-alanine-- R -lactate ligase VanA
Enterococcus faecium TX0082 GCF 000148025.1 WP 001079843.1 MULTISPECIES D-alanine-- R -lactate ligase VanA
Enterococcus faecium TX0133a01 GCF 000148325.1 WP 001079843.1 MULTISPECIES D-alanine-- R -lactate ligase VanA
Enterococcus faecium TX0133C GCF 000147315.1 WP 001079843.1 MULTISPECIES D-alanine-- R -lactate ligase VanA
Staphylococcus aureus subsp. aureus VRS2 GCF 000262835.1 WP 001079843.1 MULTISPECIES D-alanine-- R -lactate ligase VanA
Staphylococcus aureus subsp. aureus VRS3a GCF 000262855.1 WP 001079843.1 MULTISPECIES D-alanine-- R -lactate ligase VanA
Enterococcus faecium 503 GCF 000295055.2 WP 001079843.1 MULTISPECIES D-alanine-- R -lactate ligase VanA
Enterococcus faecium 506 GCF 000295495.2 WP 001079843.1 MULTISPECIES D-alanine-- R -lactate ligase VanA
Enterococcus faecium C1904 GCF 000295315.2 WP 001079843.1 MULTISPECIES D-alanine-- R -lactate ligase VanA
Enterococcus faecium C497 GCF 000295375.2 WP 001079843.1 MULTISPECIES D-alanine-- R -lactate ligase VanA
Enterococcus faecium C621 GCF 000295395.2 WP 001079843.1 MULTISPECIES D-alanine-- R -lactate ligase VanA
Enterococcus faecium E417 GCF 000295415.2 WP 001079843.1 MULTISPECIES D-alanine-- R -lactate ligase VanA
Enterococcus faecium E422 GCF 000295455.2 WP 001079843.1 MULTISPECIES D-alanine-- R -lactate ligase VanA
Enterococcus faecium ERV1 GCF 000295435.2 WP 001079843.1 MULTISPECIES D-alanine-- R -lactate ligase VanA
Enterococcus faecium ERV102 GCF 000295355.2 WP 001079843.1 MULTISPECIES D-alanine-- R -lactate ligase VanA
Enterococcus faecium ERV161 GCF 000295255.2 WP 001079843.1 MULTISPECIES D-alanine-- R -lactate ligase VanA
Enterococcus faecium ERV165 GCF 000295235.2 WP 001079843.1 MULTISPECIES D-alanine-- R -lactate ligase VanA
Enterococcus faecium ERV168 GCF 000295075.2 WP 001079843.1 MULTISPECIES D-alanine-- R -lactate ligase VanA
Enterococcus faecium ERV26 GCF 000295115.2 WP 001079843.1 MULTISPECIES D-alanine-- R -lactate ligase VanA
Enterococcus faecium ERV38 GCF 000295215.2 WP 001079843.1 MULTISPECIES D-alanine-- R -lactate ligase VanA
Enterococcus faecium ERV99 GCF 000295175.2 WP 001079843.1 MULTISPECIES D-alanine-- R -lactate ligase VanA
Enterococcus faecium P1123 GCF 000295155.2 WP 001079843.1 MULTISPECIES D-alanine-- R -lactate ligase VanA
Enterococcus faecium P1137 GCF 000295135.2 WP 001079843.1 MULTISPECIES D-alanine-- R -lactate ligase VanA
Enterococcus faecium P1139 GCF 000295035.2 WP 001079843.1 MULTISPECIES D-alanine-- R -lactate ligase VanA
Enterococcus faecium P1140 GCF 000295015.1 WP 001079843.1 MULTISPECIES D-alanine-- R -lactate ligase VanA
Enterococcus faecium P1190 GCF 000294995.2 WP 001079843.1 MULTISPECIES D-alanine-- R -lactate ligase VanA
Enterococcus faecium P1986 GCF 000294975.2 WP 001079843.1 MULTISPECIES D-alanine-- R -lactate ligase VanA
Enterococcus faecium R446 GCF 000294955.2 WP 001079843.1 MULTISPECIES D-alanine-- R -lactate ligase VanA
Enterococcus faecium R499 GCF 000294875.2 WP 001079843.1 MULTISPECIES D-alanine-- R -lactate ligase VanA
Enterococcus faecium R501 GCF 000294855.2 WP 001079843.1 MULTISPECIES D-alanine-- R -lactate ligase VanA
Enterococcus faecium S447 GCF 000294835.2 WP 001079843.1 MULTISPECIES D-alanine-- R -lactate ligase VanA
Enterococcus faecium EnGen0034 GCF 000322205.1 WP 001079843.1 MULTISPECIES D-alanine-- R -lactate ligase VanA
Enterococcus faecium EnGen0375 GCF 000407065.1 WP 001079843.1 MULTISPECIES D-alanine-- R -lactate ligase VanA
Enterococcus faecium EnGen0375 GCF 000407625.1 WP 001079843.1 MULTISPECIES D-alanine-- R -lactate ligase VanA
Enterococcus faecium EnGen0376 GCF 000407325.1 WP 001079843.1 MULTISPECIES D-alanine-- R -lactate ligase VanA
Enterococcus faecium EnGen0377 GCF 000407105.1 WP 001079843.1 MULTISPECIES D-alanine-- R -lactate ligase VanA
Enterococcus faecium EnGen0377 GCF 000407345.1 WP 001079843.1 MULTISPECIES D-alanine-- R -lactate ligase VanA
Enterococcus faecalis EnGen0253 GCF 000396865.1 WP 001079843.1 MULTISPECIES D-alanine-- R -lactate ligase VanA
Enterococcus faecium EnGen0254 GCF 000396885.1 WP 001079843.1 MULTISPECIES D-alanine-- R -lactate ligase VanA
Enterococcus faecalis EnGen0232 GCF 000396905.1 WP 001079843.1 MULTISPECIES D-alanine-- R -lactate ligase VanA
Enterococcus faecium EnGen0255 GCF 000396925.1 WP 001079843.1 MULTISPECIES D-alanine-- R -lactate ligase VanA
Enterococcus faecium EnGen0256 GCF 000396945.1 WP 001079843.1 MULTISPECIES D-alanine-- R -lactate ligase VanA
Enterococcus faecium EnGen0322 GCF 000394595.1 WP 001079843.1 MULTISPECIES D-alanine-- R -lactate ligase VanA
Enterococcus faecium EnGen0314 GCF 000394635.1 WP 001079843.1 MULTISPECIES D-alanine-- R -lactate ligase VanA
Enterococcus faecium EnGen0316 GCF 000394655.1 WP 001079843.1 MULTISPECIES D-alanine-- R -lactate ligase VanA
Enterococcus faecium EnGen0319 GCF 000394695.1 WP 001079843.1 MULTISPECIES D-alanine-- R -lactate ligase VanA
Enterococcus faecium EnGen0323 GCF 000394735.1 WP 001079843.1 MULTISPECIES D-alanine-- R -lactate ligase VanA
Enterococcus faecalis EnGen0358 GCF 000393235.1 WP 001079843.1 MULTISPECIES D-alanine-- R -lactate ligase VanA
Enterococcus faecium E155 GCF 000442045.1 WP 001079843.1 MULTISPECIES D-alanine-- R -lactate ligase VanA
Enterococcus faecium MRSN 11639 GCF 000982025.1 WP 001079843.1 MULTISPECIES D-alanine-- R -lactate ligase VanA
Enterococcus faecium MRSN 3418 GCF 000981945.1 WP 001079843.1 MULTISPECIES D-alanine-- R -lactate ligase VanA
Enterococcus faecium 17282 GCF 009846615.2 WP 001079843.1 MULTISPECIES D-alanine-- R -lactate ligase VanA
Enterococcus faecium 2605 GCF 009846555.2 WP 001079843.1 MULTISPECIES D-alanine-- R -lactate ligase VanA
Enterococcus faecium 6066 GCF 009846545.2 WP 001079843.1 MULTISPECIES D-alanine-- R -lactate ligase VanA
Enterococcus faecium 232 GCF 009846515.2 WP 001079843.1 MULTISPECIES D-alanine-- R -lactate ligase VanA
Enterococcus sp. HMSC34G12 GCF 001838605.1 WP 001079843.1 MULTISPECIES D-alanine-- R -lactate ligase VanA
Enterococcus sp. HMSC074F07 GCF 001837845.1 WP 001079843.1 MULTISPECIES D-alanine-- R -lactate ligase VanA
Enterococcus sp. HMSC072G01 GCF 001809135.1 WP 001079843.1 MULTISPECIES D-alanine-- R -lactate ligase VanA
Enterococcus sp. HMSC056C08 GCF 001809145.1 WP 001079843.1 MULTISPECIES D-alanine-- R -lactate ligase VanA
Enterococcus sp. HMSC034B11 GCF 001835885.1 WP 001079843.1 MULTISPECIES D-alanine-- R -lactate ligase VanA
Enterococcus sp. HMSC060D09 GCF 001836575.1 WP 001079843.1 MULTISPECIES D-alanine-- R -lactate ligase VanA
Enterococcus sp. HMSC077E04 GCF 001809335.1 WP 001079843.1 MULTISPECIES D-alanine-- R -lactate ligase VanA
Enterococcus sp. HMSC060D07 GCF 001836555.1 WP 001079843.1 MULTISPECIES D-alanine-- R -lactate ligase VanA
Enterococcus sp. HMSC072D11 GCF 001837695.1 WP 001079843.1 MULTISPECIES D-alanine-- R -lactate ligase VanA
Enterococcus sp. HMSC070F12 GCF 001837605.1 WP 001079843.1 MULTISPECIES D-alanine-- R -lactate ligase VanA
Enterococcus sp. HMSC073E07 GCF 001810595.1 WP 001079843.1 MULTISPECIES D-alanine-- R -lactate ligase VanA
Enterococcus sp. HMSC058D07 GCF 001810855.1 WP 001079843.1 MULTISPECIES D-alanine-- R -lactate ligase VanA
Enterococcus sp. HMSC055G03 GCF 001811575.1 WP 001079843.1 MULTISPECIES D-alanine-- R -lactate ligase VanA
Enterococcus sp. HMSC073E08 GCF 001811715.1 WP 001079843.1 MULTISPECIES D-alanine-- R -lactate ligase VanA
Enterococcus sp. HMSC077E07 GCF 001811925.1 WP 001079843.1 MULTISPECIES D-alanine-- R -lactate ligase VanA
Enterococcus sp. HMSC072F07 GCF 001812645.1 WP 001079843.1 MULTISPECIES D-alanine-- R -lactate ligase VanA
Enterococcus sp. HMSC063C12 GCF 001814315.1 WP 001079843.1 MULTISPECIES D-alanine-- R -lactate ligase VanA
Enterococcus sp. HMSC060E05 GCF 001814465.1 WP 001079843.1 MULTISPECIES D-alanine-- R -lactate ligase VanA
Enterococcus sp. HMSC066C04 GCF 001815025.1 WP 001079843.1 MULTISPECIES D-alanine-- R -lactate ligase VanA
Enterococcus sp. HMSC065H03 GCF 001815065.1 WP 001079843.1 MULTISPECIES D-alanine-- R -lactate ligase VanA
Enterococcus sp. HMSC076D08 GCF 001815195.1 WP 001079843.1 MULTISPECIES D-alanine-- R -lactate ligase VanA
Enterococcus sp. S109 ASV 20 GCF 018920165.1 WP 001079843.1 MULTISPECIES D-alanine-- R -lactate ligase VanA
Enterococcus sp. S113 ASV 20 GCF 018920085.1 WP 001079843.1 MULTISPECIES D-alanine-- R -lactate ligase VanA
Enterococcus sp. S117 ASV 20 GCF 018920105.1 WP 001079843.1 MULTISPECIES D-alanine-- R -lactate ligase VanA
Enterococcus sp. S125 ASV 20 GCF 018920045.1 WP 001079843.1 MULTISPECIES D-alanine-- R -lactate ligase VanA
Enterococcus sp. S129 ASV 20 GCF 018920005.1 WP 001079843.1 MULTISPECIES D-alanine-- R -lactate ligase VanA
Enterococcus sp. S133 ASV 20 GCF 018919965.1 WP 001079843.1 MULTISPECIES D-alanine-- R -lactate ligase VanA
Enterococcus sp. S141 ASV 20 GCF 018919995.1 WP 001079843.1 MULTISPECIES D-alanine-- R -lactate ligase VanA
Enterococcus sp. S145 ASV 20 GCF 018919945.1 WP 001079843.1 MULTISPECIES D-alanine-- R -lactate ligase VanA
Enterococcus sp. S149 ASV 20 GCF 018919875.1 WP 001079843.1 MULTISPECIES D-alanine-- R -lactate ligase VanA
Enterococcus sp. S153 ASV 20 GCF 018919905.1 WP 001079843.1 MULTISPECIES D-alanine-- R -lactate ligase VanA
Enterococcus sp. S157 ASV 20 GCF 018919925.1 WP 001079843.1 MULTISPECIES D-alanine-- R -lactate ligase VanA
Enterococcus sp. S159 ASV 20 GCF 018919865.1 WP 001079843.1 MULTISPECIES D-alanine-- R -lactate ligase VanA
Enterococcus sp. S161 ASV 20 GCF 018919835.1 WP 001079843.1 MULTISPECIES D-alanine-- R -lactate ligase VanA
Enterococcus sp. S163 ASV 20 GCF 018919805.1 WP 001079843.1 MULTISPECIES D-alanine-- R -lactate ligase VanA
Enterococcus sp. S165 ASV 20 GCF 018919815.1 WP 001079843.1 MULTISPECIES D-alanine-- R -lactate ligase VanA
Enterococcus sp. S171 ASV 20 GCF 018919785.1 WP 001079843.1 MULTISPECIES D-alanine-- R -lactate ligase VanA
Enterococcus sp. S173 ASV 20 GCF 018919765.1 WP 001079843.1 MULTISPECIES D-alanine-- R -lactate ligase VanA
Enterococcus sp. S183 ASV 20 GCF 018919685.1 WP 001079843.1 MULTISPECIES D-alanine-- R -lactate ligase VanA
